# Proline synthesis in developing microspores is required for pollen development and fertility

**DOI:** 10.1186/s12870-018-1571-3

**Published:** 2018-12-17

**Authors:** Roberto Mattioli, Marco Biancucci, Amira El Shall, Luciana Mosca, Paolo Costantino, Dietmar Funck, Maurizio Trovato

**Affiliations:** 1 Department of Biology and Biotechnology, Sapienza University of Rome, P.le A. Moro 5, 00185 Rome, Italy; 2 Department of Bioscience, University of Milan, Milan, Italy; 3 Department of Biochemical Sciences, Sapienza University of Rome, Rome, Italy; 4 Department of Biology, University of Konstanz, Universitätsstraße 10, 78464 Konstanz, Germany

**Keywords:** Proline biosynthesis, Pollen development, Tissue specificity, Anthers, Arabidopsis

## Abstract

**Background:**

In many plants, the amino acid proline is strongly accumulated in pollen and disruption of proline synthesis caused abortion of microspore development in Arabidopsis. So far, it was unclear whether local biosynthesis or transport of proline determines the success of fertile pollen development.

**Results:**

We analyzed the expression pattern of the proline biosynthetic genes *PYRROLINE-5-CARBOXYLATE SYNTHETASE 1 & 2 (P5CS1* & *2*) in Arabidopsis anthers and both isoforms were strongly expressed in developing microspores and pollen grains but only inconsistently in surrounding sporophytic tissues. We introduced in a *p5cs1/p5cs1 p5cs2/P5CS2* mutant background an additional copy of *P5CS2* under the control of the Cauliflower Mosaic Virus (CaMV*) 35S* promoter, the tapetum-specific *LIPID TRANSFER PROTEIN 12* (*Ltp12*) promoter or the pollen-specific *At5g17340* promoter to determine in which site proline biosynthesis can restore the fertility of proline-deficient microspores. The specificity of these promoters was confirmed by β-glucuronidase (GUS) analysis, and by direct proline measurement in pollen grains and stage-9/10 anthers. Expression of *P5CS2* under control of the *At5g17340* promoter fully rescued proline content and normal morphology and fertility of mutant pollen. In contrast, expression of *P5CS2* driven by either the *Ltp12* or *CaMV35S* promoter caused only partial restoration of pollen development with little effect on pollen fertility.

**Conclusions:**

Overall, our results indicate that proline transport is not able to fulfill the demand of the cells of the male germ line. Pollen development and fertility depend on local proline biosynthesis during late stages of microspore development and in mature pollen grains.

**Electronic supplementary material:**

The online version of this article (10.1186/s12870-018-1571-3) contains supplementary material, which is available to authorized users.

## Background

The importance of proline for pollen fertility has been recently highlighted by the observation that disruption of proline synthesis in Arabidopsis caused infertility by abortion during gametophyte development [1–3]. These findings provided a functional link between fertility and the accumulation of high levels of free proline under non-stressed conditions in floral organs - particularly anthers and pollen grains - of different plant species [4–10]. However, it remained to be clarified whether this high level of proline in pollen is due to local synthesis or derives from import from other, sporophytic tissues.

In higher plants, proline is synthesized via a short pathway, which catalyzes the ATP- and NADPH-consuming reduction of glutamate to proline. In the first, rate-limiting step, glutamate is converted into glutamic semialdehyde by the bifunctional enzyme Δ^1^-pyrroline-5-carboxylate synthetase (P5CS). In the second step, glutamic semialdehyde spontaneously cyclizes to Δ^1^-pyrroline-5-carboxylate (P5C), which is further reduced to proline by the enzyme P5C reductase (P5CR). Some authors postulated an alternative route for proline synthesis from ornithine, catalyzed by the sequential actions of the enzymes ornithine-δ-amino-transferase and P5CR [11, 12]. The differential localization of these two enzymes and the observation that functional *P5CS* expression is essential for reproduction (see below) suggested that synthesis from glutamate is the only functional pathway for proline biosynthesis in Arabidopsis [2, 3, 13].

In Arabidopsis, P5CS is encoded by the two paralogous genes *P5CS1* (*At2g39800*) and *P5CS2* (*At3g55610*) [14]. *P5CS1* is thought to be responsible for stress-induced proline accumulation, as homozygous *p5cs1* mutants did not accumulate proline under stress [9, 15]. In contrast, *P5CS2* is mostly involved in developmental processes such as embryo development and floral transition: *p5cs2* homozygous mutants are usually embryo lethal but can be rescued with external proline and can produce viable seeds under favorable conditions [2, 8, 15]. Rescue of *p5cs1/p5cs2* double mutants has not been reported so far and quasi-double mutants homozygous for *p5cs1* and heterozygous for *p5cs2* (*p5cs1/p5cs1 p5cs2/P5CS2*, from here on referred to as *p5cs* sesquimutants) had very low levels of free proline in vegetative tissue, were late-flowering and showed reduced male fertility. In anthers of *p5cs* sesquimutant plants, about half of the pollen grains are small, shriveled, devoid of nuclei and non-viable, as judged by Alexander staining [3]. When *p5cs* sesquimutants were allowed to self-fertilize, the transmission of the *p5cs2* mutant allele to the next generation was lower than expected and formation of abortive homozygous *p5cs2* mutant embryos was not observed. Transmission of the *p5cs2* mutant allele from pollen of *p5cs* sesquimutants to wildtype pistils was almost never observed (0 to 0.8% of observed transmission against 50% of expected transmission) indicating that only pollen with a functional *P5CS* allele developed normally and was fertile [2, 3].

Development of the male germline has been characterized in detail and divided into 13 stages [16]: Stages 1 to 7 comprise the development of sporogenous cells, meiosis and the release of free microspore tetrads inside anther locules. In stages 8 and 9 individual microspores are released from callose-encased tetrads and become vacuolated. From stage 10 onwards, both the microspore and the tapetum cells contribute to the formation of the pollen wall and exine. Stage 11 and 12 are marked by mitotic divisions giving rise to bi- and tri-cellular pollen, respectively. Stage 12 also prepares the release of the pollen grains by septum degeneration, which is completed in stage 13 with stomium rupture. In early stages, the sporogenous cells and the surrounding tapetum cells are interconnected by plasmodesmata, allowing the symplastic import of nutrients and macromolecules into the pollen mother cells [17]. The tapetum plays an essential role in pollen development and fertility as first demonstrated by genetic ablation of the tapetal cells in tobacco, which resulted in male sterility [18]. Later, Yang et al. (2003) [19] showed that a knock-out mutation of *TAPETUM DETERMINANT 1* causes male-sterility in Arabidopsis by forcing tapetal cell precursors to differentiate into microsporocytes instead of tapetum. More recently, an essential function in the development of fertile pollen has also been demonstrated for the middle layer of the anther wall [20]. During meiosis, a thick callose wall is formed between and around the microspore tetrads, which become symplasmically isolated at this stage [17, 21]. Accordingly, microspores depend on either apoplastic transport or endogenous synthesis for further accumulation of metabolites such as proline.

Available data suggest, but not demonstrate, that proline can be either synthesized in pollen grains or in surrounding sporophytic tissues. Indeed, microarray data indicate that all the genes involved in proline synthesis are expressed in developing Arabidopsis microspores and pollen grains as well as in anthers and flower buds [22, 23]. Unfortunately, most microarrays used to analyze different parts of flowers do not distinguish between *P5CS1* and *P5CS2*. We found one experiment (Gene Expression Omnibus accession GSM159352), in which gene-specific probes on a CATMA array yielded signals 2- to 10-times above background in both Col-0 and Ws-0 pollen [22, 24]. Székely et al. (2008) detected a P5CS1:GFP fusion protein in the pollen of Arabidopsis, an observation compatible either with transcription of *P5CS1* in the male germline or with the import of P5CS1 protein or mRNA. The signal of P5CS2:GFP in anthers was less clear and appeared stronger in sporophytic anther tissues [15].

On the other hand, circumstantial evidence suggested that proline can also be actively transported from distant tissues or can be released by sporophytic anther tissues to be subsequently imported into pollen grains. The long distance transport of proline through phloem vessels from vegetative to floral tissues has been documented [25, 26] and the gene *PROLINE TRANSPORTER 1 (ProT1*; *At2g39890*) coding for a specific proline carrier in Arabidopsis has been shown to be highly expressed in mature pollen [27], both evidences apparently suggesting transport as the cause of accumulation of proline in pollen grains. However, none of the single, double and triple knock-out mutants of the genes belonging to the *ProT* family (*At2g39890*, *At3g55740*, *At2g36590*) exhibited alterations either in proline content or in pollen germination efficiency [27]. Expression of further amino acid carriers with broader substrate specificity, such as *LYSINE HISTIDINE TRANSPORTER 2 (LHT2*; *At1g24400*) and *LHT4* (*At1g47670*), was detected in developing or mature pollen, but there is currently no information about the physiological function of these transporters in pollen [28].

To assess whether, and to which extent, the proline required for pollen fertility is locally synthesized in the male germ line or comes from surrounding sporophytic tissues, we analyzed the pattern of expression of *P5CS1* and *P5CS2* in Arabidopsis anthers, and generated and characterized *p5cs* sesquimutant plants expressing a functional copy of *P5CS2* either in vegetative tissue, in the tapetum or in developing pollen grains. Analyses of these plants show that the proline necessary for pollen development and particularly pollen fertility is mainly synthesized within developing microspores and mature pollen grains with little or no contribution from proline synthesized in the tapetum or other sporophytic tissues of the plant.

## Results

### The promoters of *P5CS1* and *P5CS2* are active in microspores and pollen grains at late stages of pollen development

**Fig. 1:**
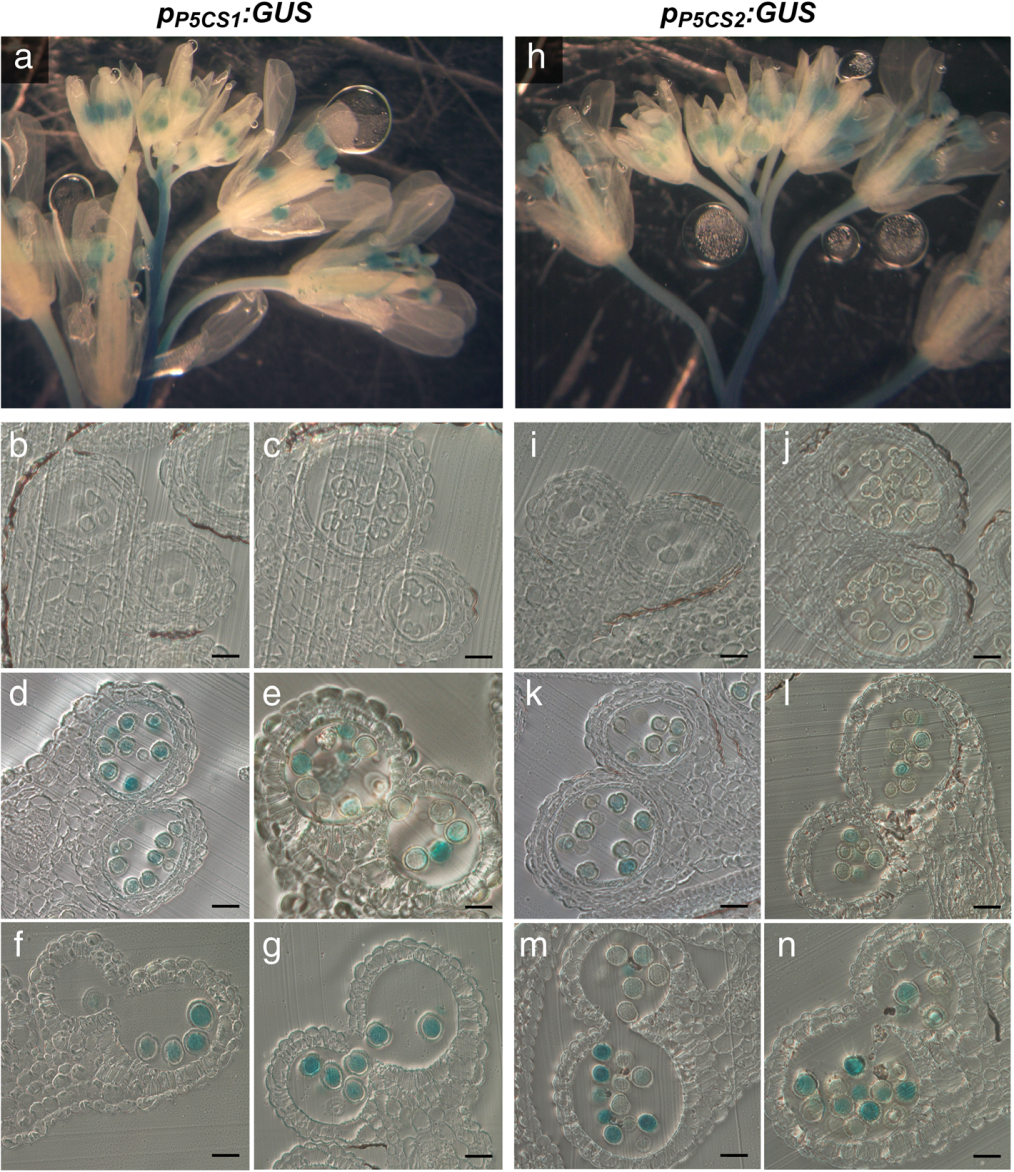
Histochemical localization of GUS activity in anthers of *p*_*P5CS1*_*:GUS* and *p*_*P5CS2*_*:GUS* transgenic Arabidopsis. Inflorescences of *p*_*P5CS1*_*:GUS* (**a-g**) and *p*_*P5CS2*_*:GUS* (**h-n**) transgenic plants were infiltrated with X-Gluc solution, stained overnight, fixed and cleared for microscopic analysis. **a, h** Whole-mount inflorescences with buds and flowers at various developmental stages showing GUS activity almost exclusively in anthers. **b-g** and **i-n** Transverse sections of anthers at different developmental stages: **b, i** Stage 8 with pre-meiotic microspore mother cells; **c, j** Stage 9 with microspore tetrads; **d, k** Stage 10 with strong activity of both *p*_*P5CS1*_*:GUS* and *p*_*P5CS2*_*:GUS* after tetrad separation; **e, l** Stage 11 with fully developed exine and degenerating tapetum; **f, m** Stage 12 with septum degeneration and **g, n** stage 13 with mature pollen at anther dehiscence. Almost no GUS staining was detected in any sporophytic anther tissue. All scale bars = 20 μm

As a first step to establish where the proline required for pollen development and fertility is synthesized, we analyzed the pattern of GUS activity under the control of the *P5CS1* and *P5CS2* promoter regions in Arabidopsis anthers at different stages of development (Fig. 1, Additional file 1: Figure S1). As promoter sequences, we used 2932 bp upstream of the *P5CS1* and 2097 bp upstream of the *P5CS2* start codon, according to Ábrahám et al. (2003) [29]. We selected and analyzed 18 independent transformants with the *p*_*P5CS1*_*:GUS* construct and 21 with *p*_*P5CS2*_*:GUS.* From each construct, we selected three homozygous lines with representative staining patterns for a more detailed analysis. Both *p*_*P5CS1*_*:GUS* and *p*_*P5CS2*_*:GUS* induced specific GUS expression in anthers, in the main stem and to a lesser extend in pedicels, but not in other parts of flowers or buds (Fig. 1a and h). Analysis of histological cross sections revealed that *p*_*P5CS1*_*:GUS* and *p*_*P5CS2*_*:GUS* were specifically active in developing microspores and mature pollen grains starting from stage 10 to stage 13 of pollen development, i.e. from immediately before the first mitotic division until the release of mature pollen (Fig. 1b, c, d, e, f and g, i, j, k, l, m and n). Expression of either fusion construct was not detected in microspores at earlier stages of development (Fig. 1b and c, i and j), or in any sporophytic tissues of the anther at any stage (Fig. 1d, and k). These results suggest that in Arabidopsis anthers proline synthesis occurs almost exclusively in male gametophytic tissues, namely microspores and pollen grains starting from stage 10, with little or no expression in surrounding sporophytic tissues, such as tapetum, middle layer and endothecium.

Bioinformatic promoter analysis, including gene ontology (GO) enrichment analysis, revealed that in the promoters of *P5CS2* and, to a lesser extent, *P5CS1* putative *cis*-regulatory elements are enriched for binding sites of transcription factors related to pollen development and fertility, pollen tube growth, anther development and double fertilization forming a zygote and endosperm (Additional file 2: Figure S2). In addition, the promoters of both *P5CS1* and *P5CS2* contain recognition sites for WRKY34 and WRKY2, well characterized transcription factors involved in pollen development and function [30, 31].

### *CaMV35S* promoter-driven sporophytic expression of *P5CS2* does not rescue the abnormalities of *p5cs1/p5cs2* pollen

**Fig. 2:**
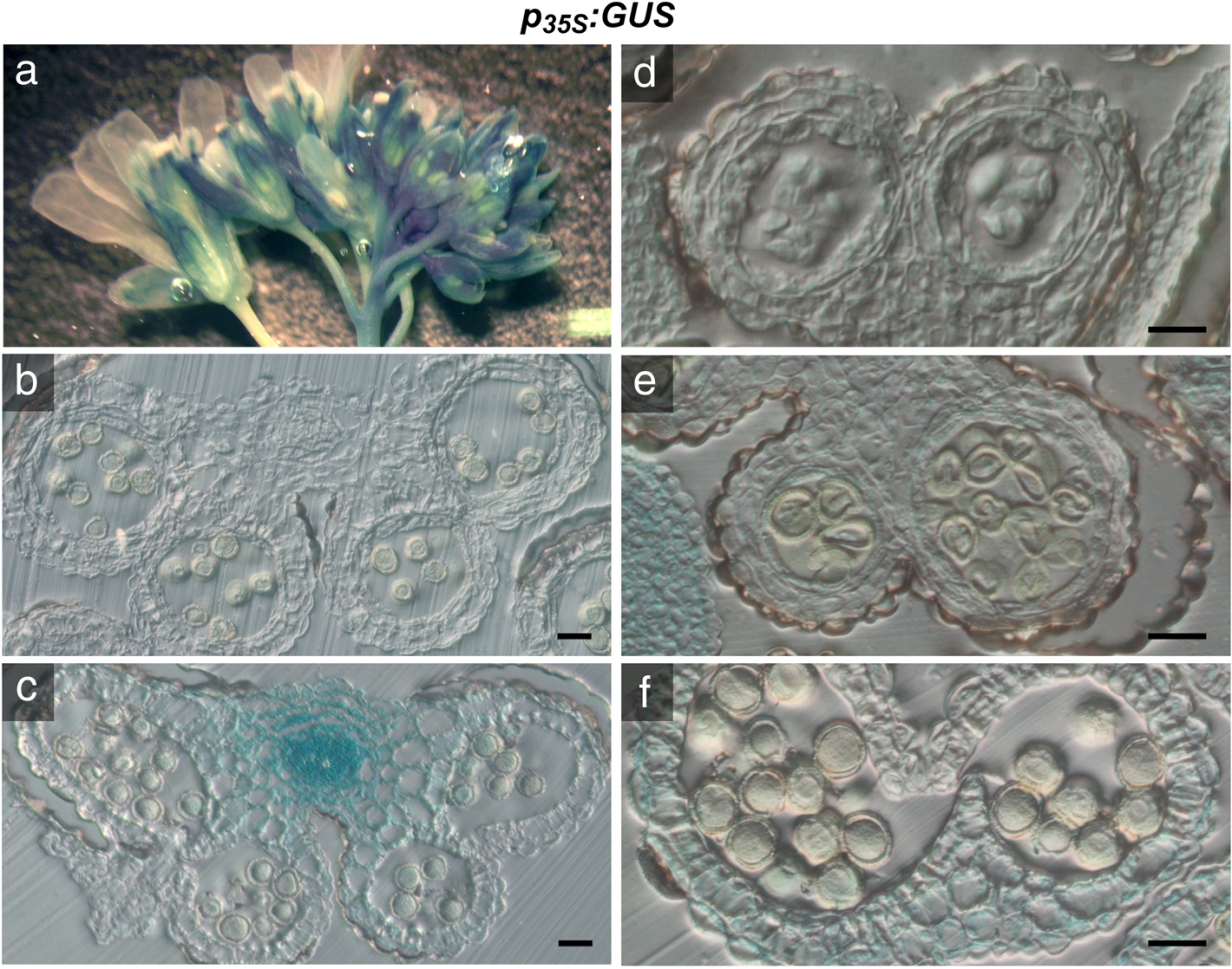
Histochemical localization of GUS activity in anthers of *p*_*35S*_*:GUS* transgenic Arabidopsis plants. Inflorescences of *p*_*35S*_*:GUS* transgenic plants were infiltrated with X-Gluc solution, stained overnight, fixed and cleared for microscopic analysis. **a** Whole inflorescences of *p*_*35S*_*:GUS* with GUS staining in filaments, sepals, pistils and in floral buds at different developmental stages. **b-f** Histological cross sections of *p*_*35S*_*:GUS* anthers: **b** unstained anther at stage 11/12 showing faint blue iridescence due to light scattering. **c** Strong GUS staining at the vascular bundle at stage 11/12. No GUS activity was detected in fully mature pollen inside the pollen sacs. Bars in (**b**) and (**c**) = 50 μm. **d** Stage 8 anther at a higher magnification showing weak, diffuse GUS staining in all sporophytic tissues. **e** Stage 9 anther with no GUS staining in uninucleate microspores. **f** Stage 12 anther with weak GUS activity in degenerating anther wall but not in mature pollen. The faint greenish color visible in some pollen grains might indicate residual activity of the 35S promoter, but could also derive from diffusion of the soluble intermediate of X-Gluc staining or a low intrinsic GUS-like activity typical of pollen grains [57–59]. Bars = 20 μm in (**d-f**)

In an attempt to complement the aberrant pollen development of *p5cs* sesquimutants by overexpression of *P5CS2*, we introduced a transgenic copy of *P5CS2* under the control of the *CaMV35S* promoter, which was reported to drive gene expression in most tissues and organs of Arabidopsis, although not in developing microspores and pollen grains [32]. To confirm the tissue-specificity of the *CaMV35S* promoter, we analyzed GUS activity in developing anthers of plants carrying a *p*_*35S*_*:GUS* construct by histochemical staining. In whole inflorescences *p*_*35S*_*:GUS* induced diffuse GUS activity in pistils, filaments and sepals as well as in floral buds at different developmental stages (Fig. 2a, Additional file 3: Figure S3A,B). Histological cross sections of anthers from *p*_*35S*_*:GUS* showed strong GUS activity in vascular bundles (Fig. 2c) and light and diffuse activity of the *CaMV35S* promoter in all sporophytic tissues of the anther and in microspores at stage 8, soon after meiotic division (Fig. 2b, c and d). At stage 9, in uninucleate microspores and in pollen grains of stage 11 and 12, no GUS activity was detected (Fig. 2e and f), confirming the results of Wilkinson et al. (1997) [32].

**Fig. 3:**
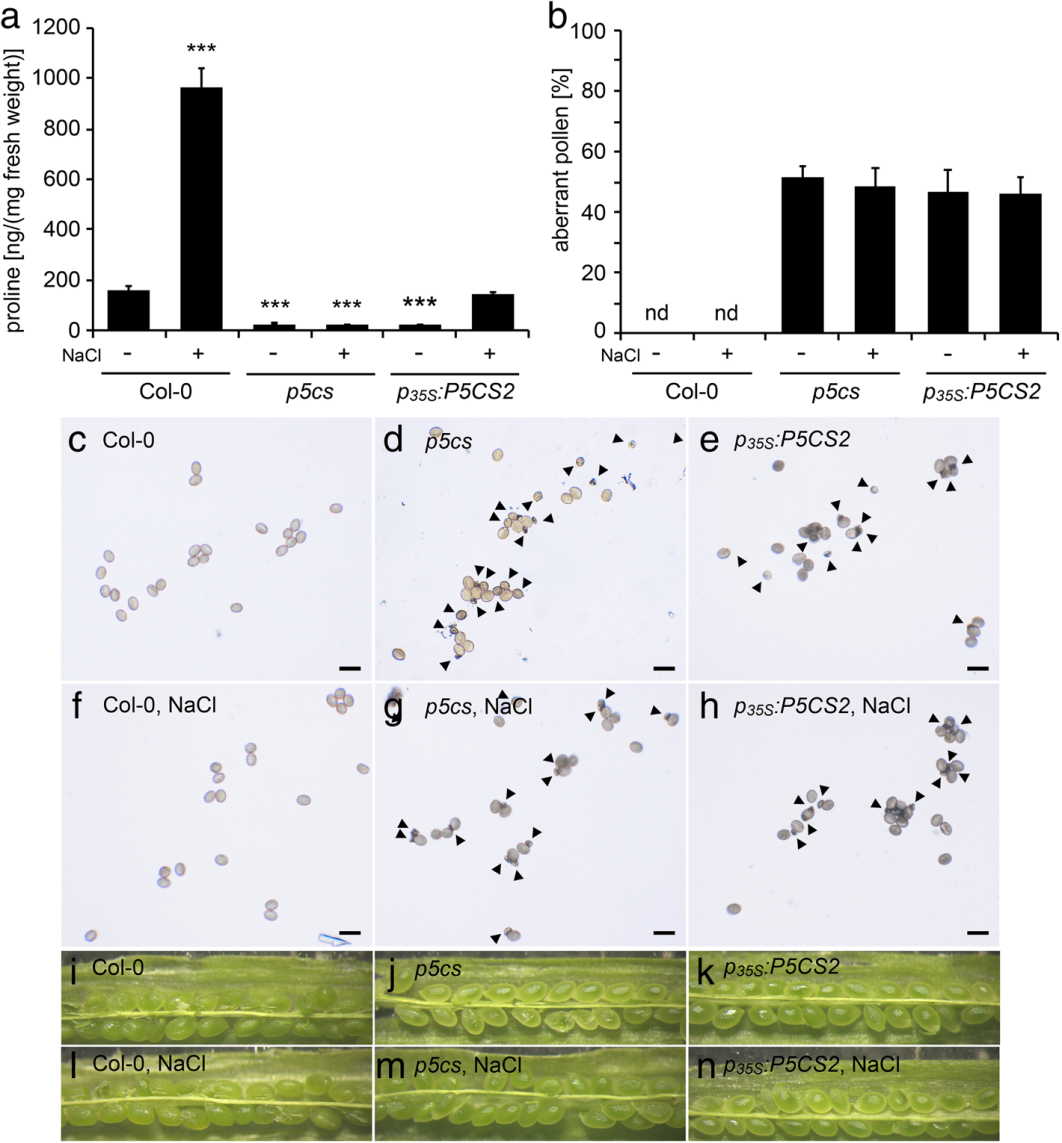
Analysis of pollen development and fertility in *p*_*35S*_*:P5CS2* lines. **a** Proline accumulation in inflorescences from wildtype (Col-0), *p5cs* sesquimutants (*p5cs*) and *p5cs* sesquimutants homozygous for the *p*_*35S*_*:P5CS2* construct (*p*_*35S*_*:P5CS2*). Plants were either watered normally or exposed to 100 mM NaCl, according to Material and Methods. Bars represent the mean ± SE of at least three samples from different plants. *** indicates significant differences from Col-0 wildtype (*p* < 0.001, by student’s T-test). **b** Percentages of aberrant pollen grains in flowers from plants treated in the same way as in (**a**). Bars represent the means of ±SE of 52 to 311 analyzed pollen grains from at least three independent plants. nd: not detected. **c-h** Bright-field microscopic pictures of pollen grains from wildtype (**c, f**), *p5cs* (**d, g**), and *p*_*35S*_*:P5CS2* (**e, h**) lines either from normally watered plants (**c, d, e**) or from plants treated with NaCl (**f, g, h**). Arrowheads indicate small, aberrant pollen grains. Bars = 50 μm. **i-n** Stereomicroscopic images of opened, premature siliques. No aberrant seeds, indicative of lethality of *p5cs2–1* homozygous embryos (compare with Fig. 6), are visible inside the siliques, neither under normal conditions (**i-k**) nor after NaCl treatment (**l-m**)

Subsequently, a *p*_*35S*_*:P5CS2* construct was introduced into *p5cs* sesquimutants to evaluate the effects of the constitutive expression of *P5CS2* in parental sporophytic tissues on the development of *p5cs1/p5cs2* double mutant pollen. However, in spite of the presence of the *p*_*35S*_*:P5CS2* construct, we detected low levels of free proline in inflorescences (Fig. 3a, Additional file 4: Table S1)*.* High levels of *P5CS2* expression and proline accumulation were observed during early stages of vegetative plant development, up to pre-flowering stage, while, in subsequent stages, both *P5CS2* expression and the level of free proline dropped to levels similar to the original *p5cs* sesquimutant line, indicating that the *p*_*35S*_*:P5CS2* transgene was silenced (data not shown). In a previous study, we observed that mild salt stress abrogated co-suppression of both endogenous *P5CS* genes induced by a transgenic *p*_*35S*_*:P5CS1* construct [9]. Accordingly, salt treatment (0.1 M NaCl) of *p5cs* sesquimutants carrying the *p*_*35S*_*:P5CS2* construct restored proline synthesis to a level similar to non-stressed wildtype plants (Fig. 3a, Additional file 4: Table S1). However, the pattern of GUS activity induced by the *p*_*35S*_*:GUS* construct in anthers was not altered by treatment with 0.1 M NaCl (data not shown).

Both under normal conditions or under mild salt stress treatment, no statistically significant differences were noticed between *p5cs* sesquimutant plants with or without the *p*_*35S*_*:P5CS2* transgene, neither in the frequency of malformed pollen (Fig. 3b, c, d, e, f, g and h) nor in the proportion of aborted embryos in siliques of self-pollinated plants (Fig. 3i, j, k, l, m and n). Furthermore, the presence of the additional *p*_*35S*_*:P5CS2* transgene did not enable the transmission of the *p5cs2–1* mutant allele to the F1 generation when wildtype pistils were cross-pollinated with *p5cs* sesquimutant pollen (Additional file 4: Table S1). It is important to notice that in spite of the identical pollen phenotype (i.e. high frequency of aberrant pollen grains and no transmission of the *p5cs2–1* allele), NaCl treatment induced a significant increase of free proline levels in anthers of *p5cs* sesquimutant plants when the *p*_*35S*_*:P5CS2* transgene was present (Fig. 3a). These findings indicate that free proline in sporophytic anther tissues at a level similar to non-stressed wildtype plants is not sufficient to complement the developmental defects of *p5cs1/p5cs2* double mutant pollen.

### Selection of tapetum- or microspore-specific promoters

**Fig. 4:**
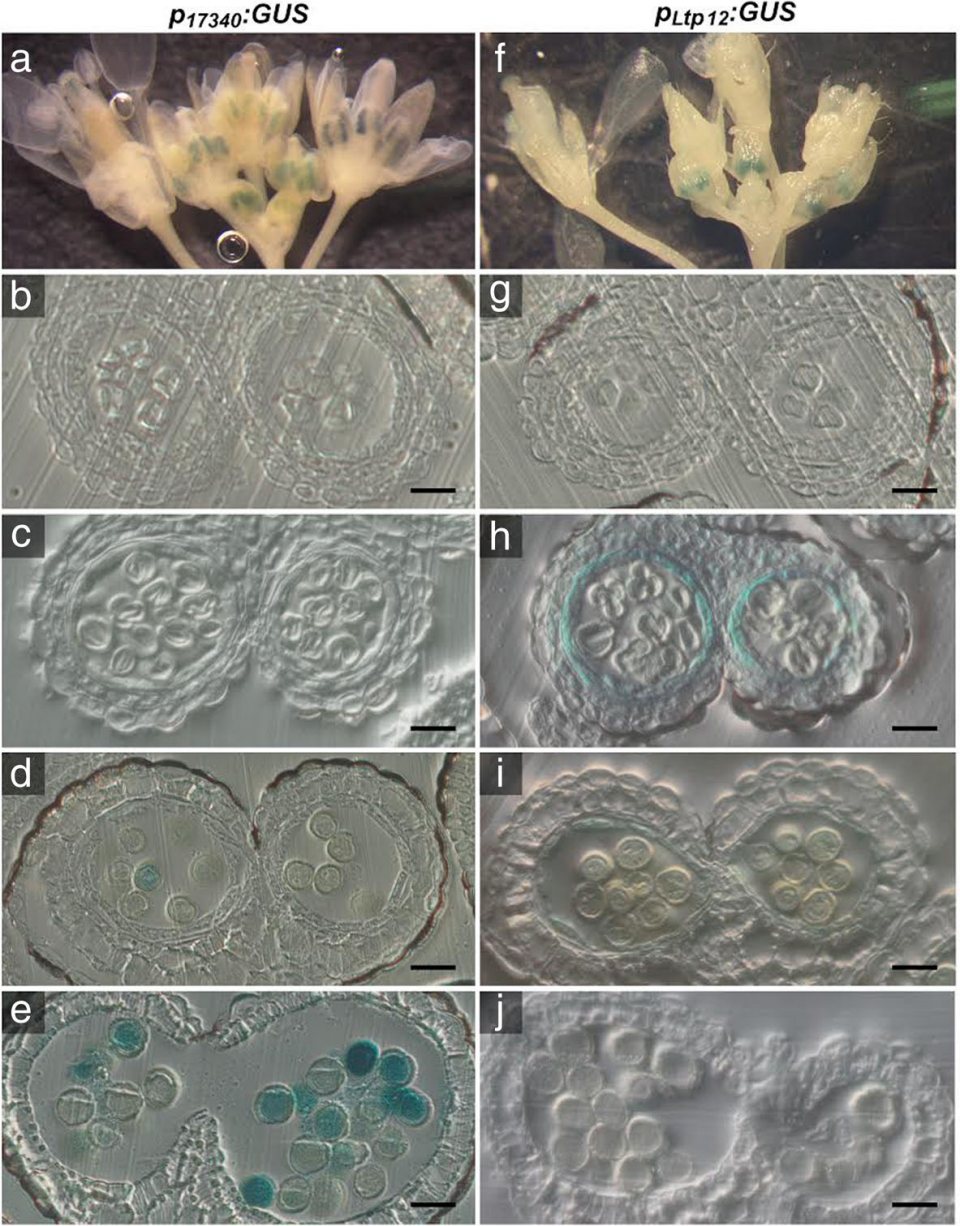
Histochemical localization of GUS activity in anthers of *p*_*17340*_*:GUS* and *p*_*Ltp12*_*:GUS* transgenic Arabidopsis plants. Inflorescences of *p*_*17340*_*:GUS* (**a-e**) and *p*_*Ltp12*_*:GUS* (**f-j**) transgenic plants were infiltrated with X-Gluc solution, stained overnight, fixed and cleared for microscopic analysis. **a, f** Whole-mount inflorescences with buds and flowers at various developmental stages showing GUS activity exclusively in anthers. **b-e** and **g-j** Transverse sections of anthers at different developmental stages: **b, g** Stage 8 with pre-meiotic microspore mother cells; **c, h** Stage 9 with microspore tetrads. A strong GUS activity is visible in the tapetum of *p*_*Ltp12*_*:GUS* (H); **d, i** Stage 10 with beginning of *p*_*17340*_*:GUS* expression in developing pollen (**d**) and residual GUS activity in the degenerating tapetum of anthers extressing the *p*_*Ltp12*_*:GUS* construct (**i**); **e, j** Stage 12 with fully developed pollen with strong GUS activity induced by *p*_*17340*_*:GUS* expression (**e**). No GUS activity was detected in stage 12 anthers of *p*_*Ltp12*_*:GUS* transgenic plants (**j**). Bars = 20 μm

To further investigate the effect of proline synthesis in the tapetum - the layer of sporophytic cells closest to the anther locule -, and in microspores and pollen grains, we decided to target *P5CS2* to these cells by the use of tissue-specific promoters. Based on available microarray and literature data, we chose the microspore- and pollen-specific promoter of *At5g17340* (*p*_*17340*_) and the tapetum-specific *Ltp12* (*At3g51590*) promoter [33–35]. The specificity of these promoters was validated by generating Arabidopsis lines harboring *p*_*17340*_*:GUS* and *p*_*Ltp12*_*:GUS* transcriptional fusion constructs and analyzing the pattern of GUS activity in anthers at different stages of development (Fig. 4, Additional file 3: Figure S3C-F). Confirming previous observations, *p*_*Ltp12*_*:GUS* induced strong GUS activity specifically in the tapetum in stages 9 and 10 of anther development, and GUS expression disappeared when the tapetum degenerated. At the same developmental stage, *p*_*17340*_*:GUS* induced strong GUS activity in microspores, which became even stronger in later stages of gametophytic development. Hardly any GUS activity was detected in other tissues of the anthers.

### Tapetum-specific expression of *P5CS2* reduces the incidence of aberrant pollen but leads to poor recovery of fertility of *p5cs1/p5cs2* pollen

**Fig. 5:**
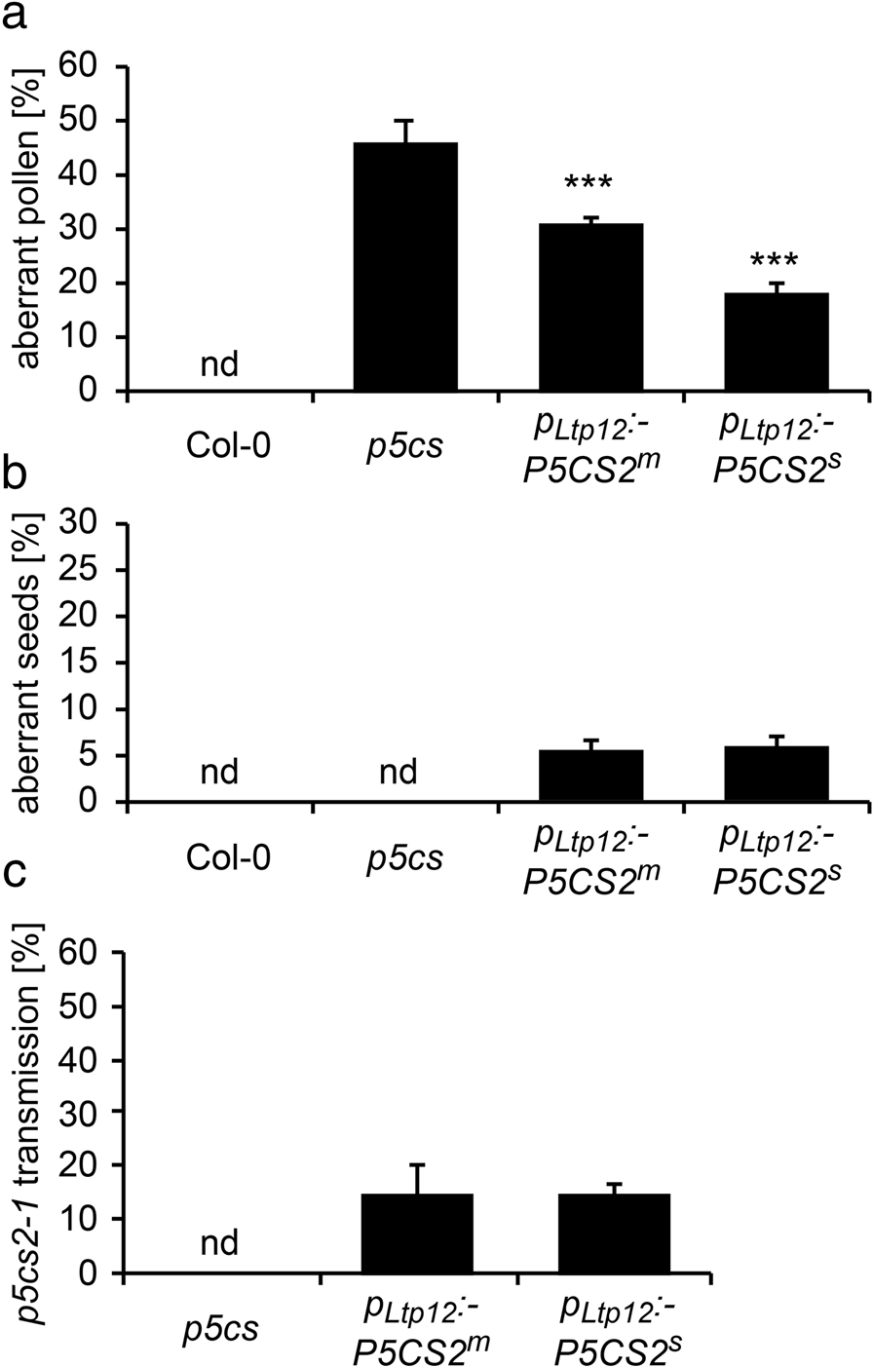
Analysis of pollen development and fertility in *p5cs* sesquimutants carrying the *p*_*Ltp12*_*:P5CS2* construct. **a** Percentages of aberrant pollen grains in wildtype (Col-0), *p5cs* sesquimutants (*p5cs*) and *p5cs* sesquimutants with a moderately (*p*_*Ltp12*_:*P5CS2*^*m*^) or strongly (*p*_*Ltp12*_*:P5CS2*^*s*^) expressed *p*_*Ltp12*_*:P5CS2* construct. nd: not detected; *** indicate significant differences from *p5cs* (*p* < 0.001, by student’s T-test). **b** Percentages of aberrant seeds in siliques after self-fertilization of the genotypes described in **a**. **c** Percentages of *p5cs2–1* mutant allele-carrying seedlings (scored by sulfadiazine resistance) obtained by cross-pollination of wildtype pistils with pollen from the plants described in **a**. Bars in **a**, **b** and **c** represent the mean ± SE of, at least, three independent experiments. nd: not detected. Data on *p*_*Ltp12*_*:P5CS2*^*s*^ represents mixed data from two independent transgenic lines

Tapetum cells provide the nutrients for microspore development and contribute to the formation of the outer layers of the pollen coat [36]. To investigate if *P5CS2* expression in the tapetum can substitute for proline synthesis in developing microspores, we introduced a *p*_*Ltp12*_*:P5CS2* construct into *p5cs* sesquimutant plants. We selected more than 20 primary transformants that carried both the *p5cs2–1* mutant allele and the *p*_*Ltp12*_*:P5CS2* transgene, of which nine carried a single copy of the transgene. In heterozygous plants, the proportion of malformed pollen was reduced compared to *p5cs* sesquimutant plants, although to a variable degree (data not shown). We selected three homozygous lines representing moderate (*p*_*Ltp12*_*:P5CS2*^*m*^) and strong (*p*_*Ltp12*_*:P5CS2*^*s*^) complementation of the pollen development defect of the *p5cs* sesquimutant for more detailed analyses (Additional file 4: Table S1). The proportion of aberrant pollen was 46 ± 3% in *p5cs* sesquimutant flowers, and this number was reduced to 31 ± 1% and 18 ± 2% in the *p*_*Ltp12*_*:P5CS2*^*m*^ and the *p*_*Ltp12*_*:P5CS2*^*s*^ lines, respectively (Fig. 5a).

Next, we determined the percentage of abortive embryos in siliques of *p*_*Ltp12*_*:P5CS2* plants. In *p5cs* sesquimutant plants, embryo-lethal homozygous individuals cannot be formed because of the infertility of pollen grains bearing the *p5cs2–1* allele, and therefore the siliques are nearly devoid of abortive embryos. In case of effective complementation of pollen fertility, 50% pollen grains bearing the *p5cs2*–*1* mutation would give rise to 25% homozygous *p5cs1/p5cs2* double mutants, which will be embryo-lethal because the *p*_*Ltp12*_*:P5CS2* does not confer P5CS expression in embryos.

When the *p*_*Ltp12*_*:P5CS2* plants were allowed to self-fertilize, the number of seeds that were aborted due to embryo development failures increased from 0% in *p5cs* sesquimutant plants to 5 ± 1% in the *p*_*Ltp12*_*:P5CS2*^*m*^ line and 6 ± 1% in the *p*_*Ltp12*_*:P5CS2*^*s*^ lines (Fig. 5b). In crosses with wildtype pistils, pollen from *p*_*Ltp12*_*:P5CS2*^*m*^ and *p*_*Ltp12*_*:P5CS2*^*s*^ plants transmitted the *p5cs2–1* mutant allele to the next generation with a frequency of approximately 15% in all three *p*_*Ltp12*_*:P5CS2* lines, which was far below the 50% expected for full complementation of the defects of *p5cs1/p5cs2* double mutant pollen (Fig. 5c). The observation of aborted embryos after selfing and the transmission of the *p5cs2–1* mutant allele via pollen in outcrossings indicated that *P5CS2* expression in the tapetum was able to restore the fertility of *p5cs1/p5cs2* double mutant pollen, although only to a rather low degree.

### Pollen-specific expression of *P5CS2* fully rescues the abnormalities of *p5cs1/p5cs2* pollen

Histochemical GUS analysis of whole inflorescences and histological cross sections of anthers confirmed that the *p*_*17340*_*:GUS* was almost exclusively active in microspores and developing pollen from stage 10 of Arabidopsis anther development (Fig. 4, Additional file 3: Figure S3E,F). Coherently, we generated a *p*_*17340*_*:P5CS2* construct to target *P5CS2* expression specifically to developing pollen and introduced it into *p5cs* sesquimutant plants to verify if the pollen-specific expression of a functional P5CS2 could rescue the morphological and functional defects of *p5cs1/p5cs2* double mutant pollen grains. The expected results for an effective complementation would be a reduction in the number of aberrant pollen grains in the anther, and an increase in the number of abortive embryos in the silique - up to 25% for full complementation.

We selected 53 kanamycin resistant transformants and among them 16 that carried the *p5cs2–1* allele, which was similar to the transmission rate observed in non-complemented *p5cs* sesquimutants. In anthers of the primary transformants, we observed between 5 and 20% aberrant pollen grains, indicating a copy-number dependent complementation by the *p*_*17340*_*:P5CS2* construct. From transformants with a single-copy insertion of the *p*_*17340*_*:P5CS2* construct, four independent homozygous lines were selected for further analyses.

**Fig. 6:**
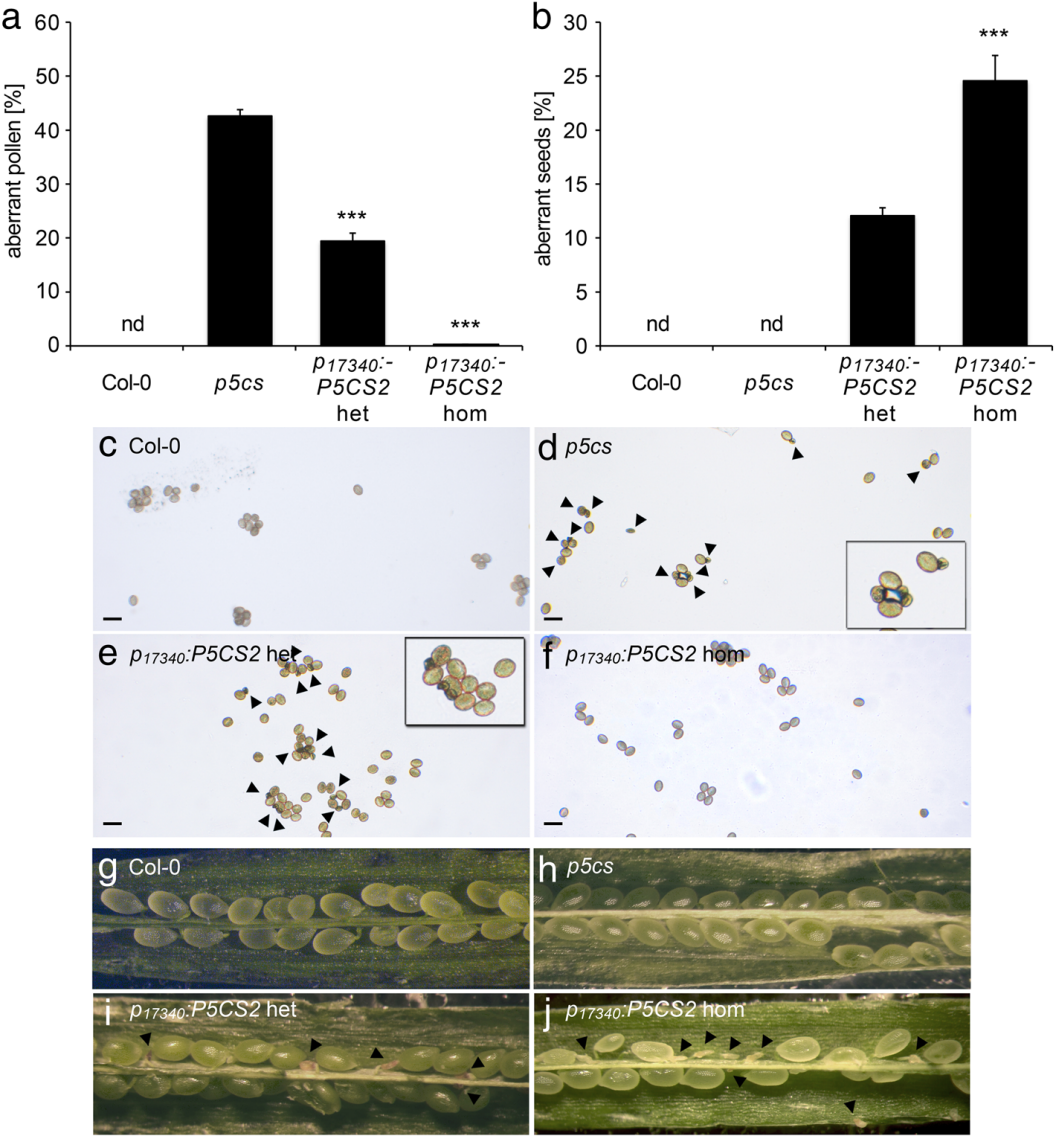
Morphological analysis of pollen and seed defects in *p5cs* sesquimutants complemented by *p*_*17340*_*:P5CS2* expression. **a** Percentages of aberrant pollen grains and **b** percentages of aberrant seeds in siliques from wildtype plants (Col-0), *p5cs* sesquimutants (*p5cs*) and heterozygous or homozygous complementation lines (*p*_*17340*_*:P5CS2* het/hom). Bars represent the means ±SE of 16–22 samples per genotype in **a** and 9 siliques per genotype in **b** mixed from plants of two independent complementation lines. nd: not detected; In **a** *** indicate significant differences from *p5cs*, while in **b** *** indicate significant differences between *p*_*17340*_*:P5CS2* het and *p*_*17340*_*:P5CS2* hom (p < 0.001, by student’s T-test). Two further complementation lines produced similar results. **c-f** Bright-field microscopic pictures of pollen grains from wildtype **c** and *p5cs* sesquimutants with **e-f** or without **d** the *p*_*17340*_*:P5CS* construct in homozygous **f** or heterozygous state **e**. An inset at higher magnification is also shown for the *p5cs* sesquimutant and the heterozygous complementation line. Arrowheads indicate small and shriveled pollen grains. **g-j** Stereomicroscopic images of opened, premature siliques from wildtype **g** and *p5cs* sesquimutants with **i-j** or without **h** the *p*_*17340*_*:P5CS* construct. Aberrant seeds indicative of lethality of *p5cs2–1* homozygous embryos are indicated by arrowheads

**Fig. 7:**
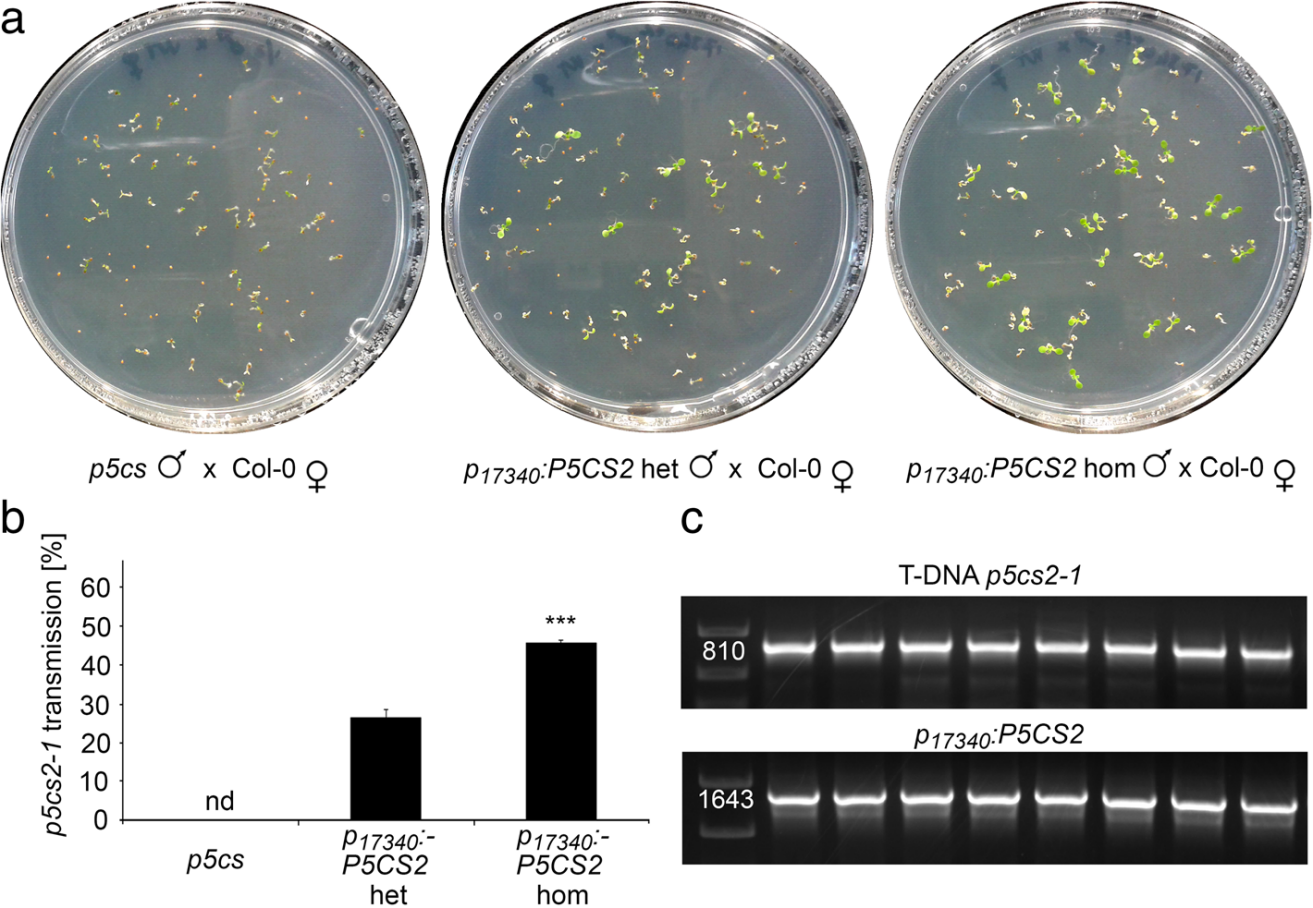
Expression of *p*_*17340*_*:P5CS2* restores fertility of *p5cs1/p5cs2* double mutant pollen. **a** Seeds produced by wildtype pistils fertilized with pollen from either a *p5cs* sesquimutant (leftmost panel), or a *p5cs* sesquimutant heterozygous (middle panel) or homozygous (rightmost panel) for the *p*_*17340*_*:P5CS2* construct were germinated on sulfadiazine-containing plates to score for the transmission of the *p5cs2–1* allele. **b** Percentages of sulfadiazine-resistant seedling among the progeny of wildtype pistils after cross-pollination as described in **a** Bars represent the mean ± SE of, at least, three independent experiments with plants of two independent transgenic lines. nd: not detected; *** indicates significant difference to *p*_*17340*_*:P5CS2* het (*p* < 0.001 by student’s T-test). Two further lines produced very similar results. **c** PCR analysis of the sulfadiazine-resistant progeny. Individual resistant plantlets were analyzed by PCR for the simultaneous presence of both the *T-DNA* generating the *p5cs2–1* mutation (upper panel) and the *p*_*17340*_*:P5CS2* construct (bottom panel). The sizes of the PCR products matched the expected numbers of base pairs (indicated alongside)

In these batches of plants, the proportion of aberrant pollen was 43 ± 1% in *p5cs* sesquimutant plants and it was strongly reduced to 20 ± 1% and 0.15 ± 0.10% in heterozygous and homozygous *p*_*17340*_*:P5CS2* transgenic plants, respectively (Fig. 6a, c, d, e and f, Additional file 4: Table S1). After self-pollination of *p*_*17340*_*:P5CS2* plants, the frequency of aborted seeds in siliques with embryos at the bent cotyledon stage was 12 ± 1% in heterozygous and 24 ± 2% in homozygous *p*_*17340*_*:P5CS2* plants (Fig. 6b, g, h, i and j). Appearance of 24 ± 2% defective embryos in homozygous *p*_*17340*_*:P5CS2* plants indicated that expression of *p*_*17340*_*:P5CS2* restored the fertility of *p5cs1/p5cs2* double mutant pollen but was not able to restore embryo development of homozygous *p5cs1*/*p5cs2* double mutant embryos. Outcrossing to wildtype pistils confirmed that heterozygous *p*_*17340*_*:P5CS2* plants produced 26 ± 2% fertile pollen with a mutant *p5cs2–1* allele (48 sulfadiazine resistant plants out of 182 in total), which was increased to 46 ± 1% when the pollen donor was homozygous for the *p*_*17340*_*:P5CS2* construct (43 sulfadiazine resistant plants out of 94; Fig. 7, Additional file 4: Table S1).

### Recovery of pollen fertility correlates with proline concentration within microspores and pollen grains

**Fig. 8:**
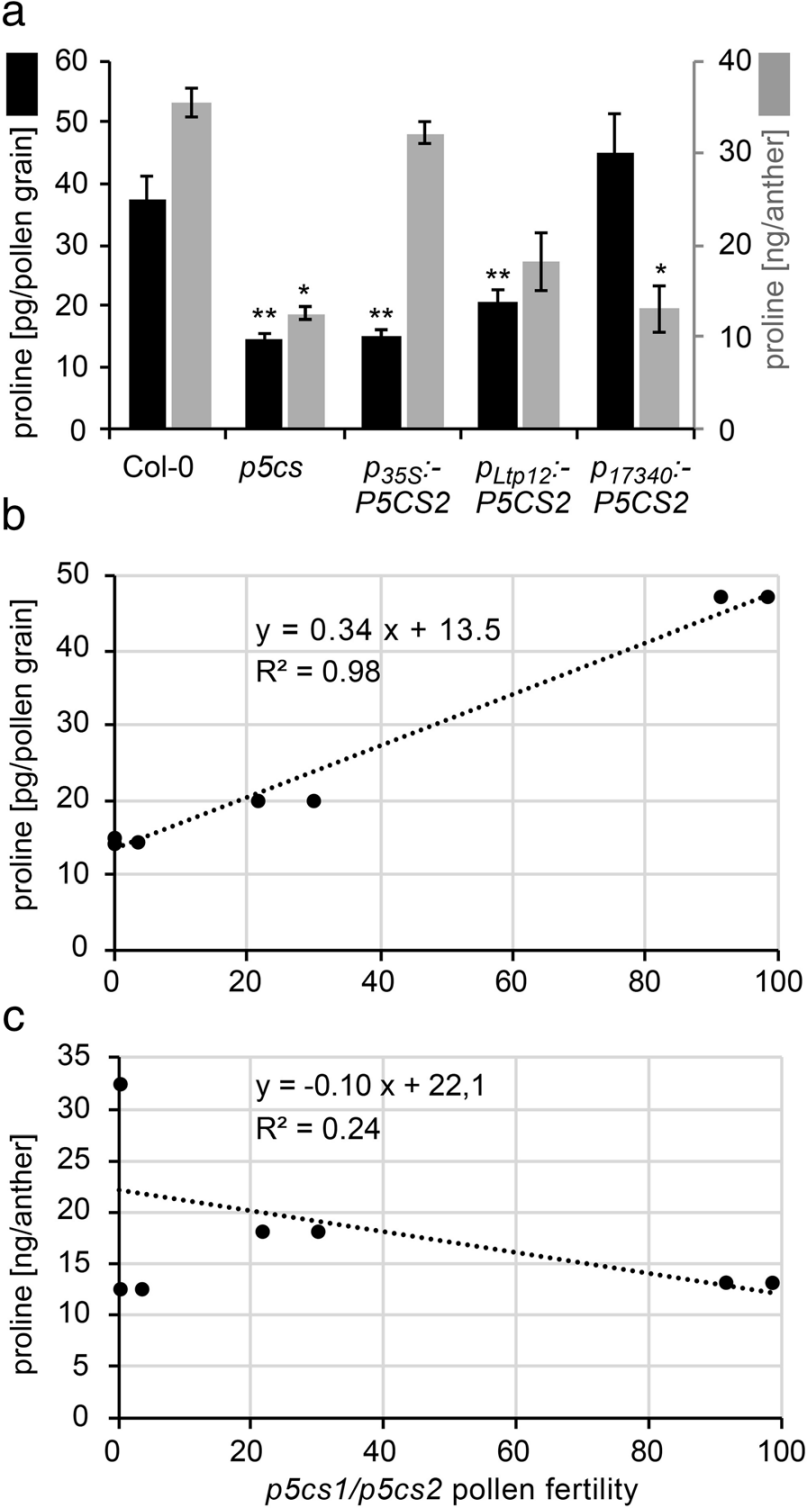
Pollen fertility correlates with proline concentration in pollen grains. **a** Proline content in pollen grains (black bars, left axis) and stage 9/10 anthers (grey bars, right axis) from wildtype (Col-0), *p5cs* sesquimutants (*p5cs*) and *p5cs* sesquimutants carrying either the *p*_*35S*_*:P5CS2, the p*_*Ltp12*_*:P5CS2*^*m*^ or the *p*_*17340*_*:P5CS2* construct. For every analysis an average of 1000 pollen grains or 200 anthers of stage 9–10 were collected and processed. Bars represent the mean ± SE of two (anthers) or three (pollen) independent samples. * and ** indicate significant differences from the corresponding Col-0 wildtype samples (*p* < 0.05 or *p* < 0.01, respectively, by student’s T-test). **b, c** Fertility of *p5cs1/p5cs2* double mutant pollen as estimated in Additional file 4: Table S1 in the different complementation lines was correlated either to the amount of proline in pollen grains **b** or to the amount of proline in anthers at stage 9–10 **c**. A strong correlation (dotted regression line) was found between proline accumulation in pollen grains and pollen fertility (R^2^ = 0.98, *P* < 0.001), while no significant correlation (dotted regression line) was found between proline accumulation in anthers of stage 9–10 and pollen fertility

To verify if tissue-specific expression of *P5CS2* indeed resulted in altered levels of proline, we measured proline content in stage 9–10 anthers and mature pollen grains of wildtype, *p5cs* sesquimutants, and *p5cs* sesquimutants transgenic for the *p*_*17340*_*:P5CS2*, *p*_*Ltp12*_*:P5CS2* or *p*_*35S*_*:P5CS2* constructs (Fig. 8a, Additional file 4: Table S1). Wildtype anthers contained 36 ± 2 ng/anther free proline and for wildtype pollen, the proline content was calculated to be 39 ± 2 pg/grain. In anthers of *p5cs2* sesquimutant plants, the proline content was approximately one third (12 ± 2 ng/anther) of the content in wildtype anthers. Averaged over normally developed and malformed pollen, the proline content in *p5cs2* sesquimutant pollen was 14 ± 1 pg/grain. Both *p*_*Ltp12*_*:P5CS2* or *p*_*35S*_*:P5CS2* caused a significant increase in the proline content in anthers, but only *p*_*Ltp12*_*:P5CS2* induced a higher proline content in pollen. Conversely, *p*_*17340*_*:P5CS2* had no effect on the proline content in stage 9/10 anthers but restored the proline content in mature pollen to the level of wildtype pollen. When compared to the fertility of *p5cs1/p5cs2* double mutant pollen in the different lines, estimated from the appearance of embryo-lethal *p5cs2–1* homozygous seeds after selfing or from the transmission rate of the *p5cs2–1* allele to wildtype pistils, proline content of pollen showed a strong positive correlation (R^2^ = 0.98, *p* < 0.001; Fig. 8b), whereas proline content in anthers did not correlate with pollen fertility (Fig. 8c).

## Discussion

Consistent with the high proline concentration found in pollen grains of different plant species [4, 7, 10, 37], proline biosynthesis has been shown to be necessary for pollen development and fertility in Arabidopsis [2, 3], but it was still unknown whether proline needs to be synthesized in developing pollen or can also be synthesized in sporophytic cells outside the pollen sac and transported into pollen grains.

### *P5CS1* and *P5CS2* are strongly expressed in developing microspores but not in sporophytic cells outside the pollen sac

A first indication of the gametophytic origin of the proline accumulated in pollen grains derived from the analysis of the promoter activity of the proline biosynthetic genes *P5CS1* and *P5CS2* in Arabidopsis flowers. Although tissue-specific, development-related expression of *P5CS1* and *P5CS2* has already been reported [15, 38], a detailed histological analysis of the expression of these genes throughout anther development was still lacking. To fill this gap, we analyzed anthers of Arabidopsis lines expressing *p*_*P5CS1*_*:GUS* or *p*_*P5CS2*_*:GUS*. Both constructs induced strong GUS expression in male gametophytic tissues but no significant expression in surrounding sporophytic tissues, which is partially in contrast to the data from Szekely et al. (2008), who observed green fluorescence induced by a *p*_*P5CS2*_*:P5CS2:GFP* construct in sporophytic anther tissue but only sporadically in pollen or precursor cells. Export of P5CS2 mRNA or protein from the gametophytic cell line into surrounding anther tissue is possible, but highly unlikely at later stages of pollen development. The strong GUS staining observed in microspores and pollen from both *p*_*P5CS1*_*:GUS* and *p*_*P5CS2*_*:GUS* plants indicates a strong activity of the *P5CS1* and *P5CS2* promoters, which is consistent with publicly available transcriptome data [22, 23, 35]. It is possible that the GUS mRNA or protein differs in stability from the Arabidopsis P5CS1 and P5CS2 proteins and thus may not entirely reflect the timing of P5CS1 and P5CS2 expression in the male germline. Proteome analyses of Arabidopsis microspores or pollen did so far not detect peptides derived from P5CS1 or P5CS2 [39–41]. However, in a recent study of tobacco pollen development, P5CS-derived peptides were detected in mature pollen and in pollen tubes but not at earlier developmental stages [42]. A metabolomic analysis of development of the tobacco male gametophyte found that proline accumulation started with pollen mitosis I and peaked in desiccated pollen, while proline concentration quickly declined during pollen tube growth [43]. In the same study, Rotsch et al. (2017) reported a similar pattern of accumulation also for pipecolic acid, a non-proteinogenic amino acid that differs from proline by one additional CH_2_-group in the ring structure.

In addition, bioinformatic identification of putative *cis*-regulatory elements in the upstream regions of *P5CS1* and *P5CS2*, which were analyzed with PlantPan 2.0 and Place, revealed an enrichment in putative binding sites for transcription factors related to pollen development and fertility. In particular, the promoter of *P5CS2* contains binding motifs for WRKY2 and WRKY34, the best-characterized transcription factors involved in pollen development and function [30, 31]. No putative binding sites for transcription factors related to tapetum development or function were identified by these programs, indicating that our localization data obtained with the *p*_*P5CS2*_*:GUS* construct is accurate.

### Only *P5CS2* overexpression in microspores and pollen grains fully recovers the abnormalities of *p5cs1/p5cs2* double mutant pollen grains

To confirm the importance of proline synthesis in gametophytic cells, we generated *p5cs* sesquimutant plants containing an additional copy of *P5CS2* expressed specifically either in gametophytic or sporophytic cells of the anther. The rationale was to assess which construct, and to what extent, could rescue the functionality of *p5cs1/p5cs2* double mutant pollen. We show that expression of *P5CS2* in microspores and pollen grains with the pollen-specific promoter of *At5g17340* resulted in full complementation of the developmental and fertility defects of *p5cs1/p5cs2* double mutant pollen. We conclude that P5CS activity inside microspores and developing gametophytes is sufficient to provide the proline needed for pollen development and fertility.

Conversely, *p5cs* sesquimutants expressing an additional copy of *P5CS2* in the tapetum (*p*_*Ltp12*_*:P5CS2)* or in sporophytic cells outside the pollen sac (*p*_*35S*_*:P5CS2)* showed only very limited or no complementation of *p5cs1/p5cs2* double mutant pollen morphology and fertility. Expression of *p*_*Ltp12*_*:P5CS2* and *p*_*35S*_*:P5CS2* significantly increased the levels of proline in anthers but had little or no effect on the proline content in pollen grains of *p5cs* sesquimutants. The observation that the *p*_*Ltp12*_*:P5CS2* construct complemented the pollen abnormalities of *p5cs* sesquimutants more efficiently than *p*_*35S*_*:P5CS2* in spite of the higher level of proline found in anthers of the latter may be explained by the different expression patterns*.* The *p*_*Ltp12*_*:P5CS2* construct induces *P5CS2* expression in the tapetum, which directly surrounds the cells of the gametophytic cell lineage. Obviously transport of proline to microspores is more efficient over short distances and might occur through plasmodesmata at early stages of microspore development or via proline leakage from tapetal cells into the extracellular space, potentially during tapetum degeneration. The capacity of developing pollen to take up extracellular proline by means of high *ProT1* expression has been demonstrated, although the absence of pollen defects in *proT* mutants suggested that it is of minor importance under wildtype conditions [27]. In contrast, proline synthesized through the activity of *p*_*35S*_*:P5CS2* in the anther vasculature does not seem to be accessible for developing pollen.

The failure of *P5CS2* expression in the tapetum or the anther vasculature to fully restore development and fertility of *p5cs1/p5cs2* double mutant pollen strongly indicates, that *P5CS* expression in the male germline is not only sufficient, but also necessary for pollen fertility. Consistently, a strong correlation between proline content in pollen and fertility was observed, whereas proline levels in anthers did not show a clear correlation with fertility of *p5cs1/p5cs2* double mutant pollen. It remains to be determined, which precursors are used for proline biosynthesis and how they are delivered to the developing pollen to ensure full fertility.

### Possible functions of proline accumulation in pollen

Several functions have been proposed for the high accumulation of proline in pollen: It may serve to maintain pollen viability despite desiccation during the transport to distant pistils, it may help the pollen to rehydrate after the arrival at the pistil and it may provide an energy source or source of building material during pollen tube growth [1, 44]. Additionally, signaling functions have been proposed for proline as well as for the structurally similar pipecolic acid, whereas so far, no functional or metabolic link between these two metabolites has been reported [45–47]. Furthemore, proline may serve as a reward for pollen dispersing insects that often deliver only a small fraction of the pollen to other flowers and use the major part as energy- and protein-rich food source.

Our data demonstrate that high levels of proline are required already for the development of fertile pollen. The misshaped and infertile *p5cs1/p5cs2* double mutant pollen of *p5cs* sesquimutants were shown to be devoid of DNA and storage compounds, indicating that a controlled cell death program is initiated during development [3]. It remains to be determined if programmed cell death is initiated specifically by the lack of proline or merely by developmental retardation of proline deficient microspores compared to proline synthesizing neighbors. In maize roots, the level of proline regulated cell cycle progression and a similar mechanism may be in place during gametophytic mitoses giving rise to trinucleate pollen [48]. *P5CS2* expression in the tapetum having a stronger effect on development than on fertility of *p5cs1/p5cs2* double mutant pollen indicated that the threshold for cell death induction might be lower than the proline level required to support full fertility.

## Conclusions

In this study, we show that most, if not all, of the effects of proline on pollen development and fertility can be accounted for by local synthesis inside developing microspores and mature pollen grains, and that the contribution of proline transport from different sporophytic tissues, if any, is very limited. Our findings open up interesting possibilities for breeding approaches: Overproduction of proline in pollen may increase pollen fertility and thus crop yield under adverse conditions, whereas inhibition of proline biosynthesis in pollen would enable conditional male sterility for hybrid production.

## Methods

### Plant growth conditions

Wildtype and mutant *Arabidopsis thaliana* (L) Heynh., ecotype Columbia-0 (Col-0), were grown in a growth chamber at 24/21 °C with light intensity of 300 μE m^− 2^ s^− 1^ under 16 h light and 8 h dark per day. An Arabidopsis line homozygous for *p5cs1* and heterozygous for *p5cs2* (referred to as *p5cs* sesquimutant), has been characterized and described previously [2, 3]. To carry out salt treatment, plants were watered twice a week with 0.1 M NaCl solution from the onset of flowering to the end of the experiment. To analyze *p5cs2–1* mutant allele transmission via pollen during cross-pollination or during selfing, surface sterilized seeds were stratified for three days at 4 °C, and germinated on ½xMS plates supplemented with 12 μg/ml sulfadiazine and 2% (*w*/*v*) sucrose. Presence of the *p5cs2–1* mutant allele was further confirmed by PCR analysis of random samples using specific primers for the *P5CS2*:T-DNA junction or the sulfadiazine resistance gene (Additional file 5: Table S2).

### Generation of transgenic plants

Molecular cloning techniques were performed according to standard protocols using primers listed in Additional file 5: Table S2. Enzymes used in this work were purchased from Thermo Fisher Scientific or New England Biolabs. RNA and DNA extractions were performed as previously described [3, 8]. The constructs *p*_*P5CS1*_*:GUS*, *p*_*P5CS2*_*:GUS* and *p*_*17340*_*:GUS* were generated by replacing the *CaMV35S* promoter in pBI121 (Clontech, Paolo Alto, California) with either the 2932 bp upstream of the start codon of *AtP5CS1*, the 2097 bp upstream of *AtP5CS2* or the 2632 bp upstream of *At5g17340*, either by direct ligation or by Gibson assembly*.* To generate *p*_*35S*_*:P5CS2* and *p*_*17340*_*:P5CS2* constructs, the GUS coding sequence was replaced by the cDNA of *AtP5CS2*. For the *p*_*Ltp12*_*:GUS* construct, the first six codons of *Ltp12* (*At3g51590*) and 1092 bp of upstream sequence were inserted into pENTR-D-TOPO and transferred to pHGWFS7 by LR recombination [49]. For the *p*_*Ltp12*_*:P5CS2* construct, the cDNA of *AtP5CS2* and was inserted into pENTR-D-TOPO and the *Ltp12* promoter region was inserted by Gibson assembly into a unique NcoI site generated at the start codon. The resulting *p*_*Ltp12*_*:P5CS2* fusion construct was transferred into pEG301 by LR recombination [50]. All constructs including the native pBI121 were introduced into wildtype Arabidopsis plants or *p5cs* sesquimutants by floral dip with *Agrobacterium tumefaciens* strain GV3101 [51]. Kanamycin, hygromycin or BASTA selection were used to isolate T1 transformants, T2 single-insertion lines, and T3 homozygous lines. Presence of the desired transgene in each line was confirmed by PCR and for each GUS or fertility analysis, homozygous plants from at least two independent lines were used.

### Assessment of pollen development and fertility

To evaluate developmental aberrations, pollen grains were tapped on a glass slide, photographed under a light microscope and the percentage of aberrant pollen grains over total pollen grains was scored. Fertility was scored by comparing the expected inheritance rate during selfing, or in crosses of mutant pollen with wildtype pistils, with the observed transmission of the *p5cs2–1* mutant allele. For the analysis of embryo development, siliques were dissected under a stereomicroscope (Zeiss Stevi SV 6, Carl Zeiss Microimaging GmbH, Jena, Germany). Digital images were acquired with a Jenoptik ProgResW C3 digital camera (Jenoptik, Jena, Germany). All analyses have been repeated at least four times. Differences between expected and observed segregation or transmission ratios were analyzed for significance with χ^2^ tests.

### Proline analysis

Proline content in seedlings or whole inflorescences was measured according to Bates [52], using L-proline as standard. Every measurement was repeated at least three times and represents the average either from more than one hundred 14-day-old seedlings or from inflorescences pooled from five plants. Proline content in anthers or pollen grains was measured by comparing HPLC chromatograms against an L-proline standard. To extract proline from pollen grains, about 10,000 pollen grains were collected from flowers on a glass slide. A representative number of microscopic sectors were photographed with an Axio Imager.A2 light microscope (Zeiss, Germany), equipped with a DC500 digital camera (Leica, Germany), and analyzed with ImageJ to estimate pollen number [53]. The pollen grains were collected from the glass slides in a suitable volume of 3% (*w*/*v*) 5-sulfosalicylic acid (~ 50 μl) and subsequently concentrated under vacuum to adjust pollen concentration to 1,000 μl^− 1^. To extract proline from anthers, about 200 anthers were collected at stage 9–10 under a dissection microscope and extracted in 50 μl of 3% (w/v) 5-sulfosalicylic acid. The developmental stages of the anthers were established as described by Cecchetti et al. (2015) [54]. The extract was centrifuged at 14,000 g for 20 min and the supernatant reduced to 7 μl under vacuum. The amino acids present in the extract were derivatized with DABS (4-N, N-dimethylaminoazobenzene-4′-sulfonyl chloride, Sigma-Aldrich, USA), according to Francioso et al. (2017) [55]. Gradient grade solvents used for chromatographic analyses were purchased from Carlo Erba Reagents (Milan, Italy). Amino acid standards and all other reagents were obtained from Sigma-Aldrich (St. Louis, MO, USA).

### Histochemical GUS staining and imaging

For analysis of GUS activity, samples were infiltrated by vacuum for 1 h with 5-bromo-4-chloro-3-indolyl-β-D-glucuronide (X-Gluc) solution supplemented with 1 mM potassium ferricyanide as an oxidative catalyst and then incubated over night at 37 °C [56]. Green tissues were then fixed and cleared under constant agitation in methanol/acetic acid (3:1, v:v) for 4 h at room temperature, followed by several washes in 70% ethanol. Plant samples for histological analysis were dehydrated, embedded in Technovit 7100 resin (Heräeus Kulzer, Wehrheim, Germany), and cut into 8 μm sections with a HM 350 SV automatic microtome (Microm, Walldorf, Germany). Histological images were acquired with an Axio Imager.A2 light microscope (Zeiss, Germany), equipped with a DC500 digital camera (Leica, Germany).

## Additional Files

Additional file 1: **Figure S1.** Histochemical localization of GUS activity in anthers of *p*_*P5CS1*_*:GUS and p*_*P5CS2*_*:GUS* transgenic Arabidopsis plants. Inflorescences of *p*_*P5CS1*_*:GUS* **(A-C)** and *p*_*P5CS2*_*:GUS* **(D-F)** transgenic plants were infiltrated with X-Gluc solution, stained overnight, fixed and cleared for microscopic analysis. Each panel shows a whole-mount anther at stage 12/13 from an independent transgenic line. GUS activity was exclusively detected in pollen grains. Scale bars are 50 μm in A, B, D, E and 25 μm in C and F. (PDF 2779 kb)

Additional file 2: **Figure S2.** Predicted *cis*-regulatory elements in the promoters of *P5CS1* and *P5CS2*. Schematic map outlining the main putative binding sites for transcription factors derived from a PlantPAN2 (http://plantpan2.itps.ncku.edu.tw) and PLACE (http://www.dna.affrc.go.jp/PLACE/) in silico analysis of *P5CS1* (*At2g39800*) and *P5CS2* (*At3g55610*) promoters. The promoter analysis was carried out on 2932 bp and 2097 bp upstream of the start codons of either *P5CS1* or *P5CS2*, respectively. Putative *cis*-regulatory elements corresponding to binding motifs of transcription factors related to pollen development and fertility (SBP, bHLH, WRKY; GO terms “associated with pollen development” [GO:0009555], “pollen tube growth” [GO:0009860], “anther development” [GO:00048643] and “double fertilization forming a zygote and endosperm” [GO:0009567]) are significantly enriched and highlighted in red. (PDF 1149 kb)

Additional file 3: **Figure S3.** Histochemical localization of GUS activity in anthers of *p*_*35S*_*:GUS*, *p*_*Ltp12*_*:GUS* and *p*_*17340*_*:GUS* transgenic Arabidopsis plants. Inflorescences of *p*_*35S*_*:GUS* **(A,B)**, *p*_*Ltp12*_*:GUS* **(C,D)** and *p*_*17340*_*:GUS* **(E,F)** transgenic plants were infiltrated with X-Gluc solution, stained overnight, fixed and cleared for microscopic analysis. Each panel shows a whole-mount anther at stage 12/13 **(A,B,E,F)** or stage 9/10 **(C,D)** from an independent transgenic line. GUS activity was detected in the filaments and vascular tissues of *p*_*35S*_*:GUS* anthers but not in pollen grains. The *p*_*Ltp12*_*:GUS* construct induced GUS activity specifically in the tapetum, whereas GUS activity in *p*_*17340*_*:GUS* transgenic anthers was almost exclusively detected in pollen grains. Scale bars are 50 μm. (PDF 2798 kb)

Additional file 4: **Table S1.** Overview of pollen morphology and fertility data. (PDF 22 kb)

Additional file 5: **Table S2.** Primers used in this study (PDF 35 kb)

## Abbreviations

CaMV35S: Cauliflower mosaic virus 35S transcript
CATMA: Complete arabidopsis transcriptome microarray
GUS: β-glucuronidase
*LHT*: *Lysine histidine transporter*
*Ltp12*: *Lipid transfer protein 12*
P5C: Δ^1^-pyrroline-5-carboxylate
P5CR: P5C reductase
P5CS: P5C synthetase
*ProT*: *Proline transporter*
X-Gluc: 5-bromo-4-chloro-3-indolyl-β-D-glucuronide.

## References

[1] Biancucci M, Mattioli R, Forlani G, Funck D, Costantino P, Trovato M (2015). Role of proline and GABA in sexual reproduction of angiosperms. Front Plant Sci.

[2] Funck D, Winter G, Baumgarten L, Forlani G (2012). Requirement of proline synthesis during Arabidopsis reproductive development. BMC Plant Biol.

[3] Mattioli R, Biancucci M, Lonoce C, Costantino P, Trovato M (2012). Proline is required for male gametophyte development in Arabidopsis. BMC Plant Biol.

[4] Chiang HH, Dandekar AM (1995). Regulation of proline accumulation in *Arabidopsis thaliana* (L) Heynh during development and in response to desiccation. Plant Cell Environ.

[5] Khoo U, Stinson HT (1957). Free amino acid differences between cytoplasmic male sterile and normal fertile anthers. Proc Natl Acad Sci U S A.

[6] Krogaard H, Andersen AS (1983). Free amino-acids of Nicotiana-Alata anthers during development Invivo. Physiol Plant.

[7] Lansac AR, Sullivan CY, Johnson BE (1996). Accumulation of free proline in sorghum (*Sorghum bicolor*) pollen. Can J Bot/Rev Can Bot.

[8] Mattioli R, Falasca G, Sabatini S, Altamura MM, Costantino P, Trovato M (2009). The proline biosynthetic genes *P5CS1* and *P5CS2* play overlapping roles in Arabidopsis flower transition but not in embryo development. Physiol Plant.

[9] Mattioli R, Marchese D, D'Angeli S, Altamura MM, Costantino P, Trovato M (2008). Modulation of intracellular proline levels affects flowering time and inflorescence architecture in Arabidopsis. Plant Mol Biol.

[10] Schwacke R, Grallath S, Breitkreuz KE, Stransky E, Stransky H, Frommer WB, Rentsch D (1999). LeProT1, a transporter for proline, glycine betaine, and gamma-amino butyric acid in tomato pollen. Plant Cell.

[11] Mestichelli LJ, Gupta RN, Spenser ID (1979). The biosynthetic route from ornithine to proline. J Biol Chem.

[12] Roosens NH, Thu TT, Iskandar HM, Jacobs M (1998). Isolation of the ornithine-delta-aminotransferase cDNA and effect of salt stress on its expression in *Arabidopsis thaliana*. Plant Physiol.

[13] Funck D, Stadelhofer B, Koch W (2008). Ornithine-δ-aminotransferase is essential for arginine catabolism but not for proline biosynthesis. BMC Plant Biol.

[14] Strizhov N, Ábrahám E, Ökrész L, Blickling S, Zilberstein A, Schell J, Koncz C, Szabados L (1997). Differential expression of two *P5CS* genes controlling proline accumulation during salt-stress requires ABA and is regulated by ABA1, ABI1 and AXR2 in Arabidopsis. Plant J.

[15] Székely G, Ábrahám E, Cséplő A, Rigó G, Zsigmond L, Csiszár J, Ayaydin F, Strizhov N, Jásik J, Schmelzer E (2008). Duplicated *P5CS* genes of Arabidopsis play distinct roles in stress regulation and developmental control of proline biosynthesis. Plant J.

[16] Bowman JL (1994). Arabidopsis : an atlas of morphology and development.

[17] Sager R, Lee J-Y (2014). Plasmodesmata in integrated cell signalling: insights from development and environmental signals and stresses. J Exp Bot.

[18] Mariani C, De Beuckeleer M, Truettner J, Leemans J, Goldberg RB (1990). Induction of male sterility in plants by a chimaeric ribonuclease gene. Nature.

[19] Yang SL, Xiea LF, Mao HZ, Puah CS, Yang WC, Jiang LX, Sundaresan V, Ye D (2003). TAPETUM DETERMINANT1 is required for cell specialization in the Arabidopsis anther. Plant Cell.

[20] Cecchetti V, Celebrin D, Napoli N, Ghelli R, Brunetti P, Costantino P, Cardarelli M (2017). An auxin maximum in the middle layer controls stamen development and pollen maturation in Arabidopsis. New Phytol.

[21] Al Mamun E, Cantrill LC, Overall RL, Sutton BG (2005). Cellular organisation in meiotic and early post-meiotic rice anthers. Cell Biol Int.

[22] Winter D, Vinegar B, Nahal H, Ammar R, Wilson GV, Provart NJ (2007). An “electronic fluorescent pictograph” browser for exploring and analyzing large-scale biological data sets. PLoS One.

[23] Hruz T, Laule O, Szabo G, Wessendorp F, Bleuler S, Oertle L, Widmayer P, Gruissem W, Zimmermann P (2008). Genevestigator v3: a reference expression database for the meta-analysis of transcriptomes. Adv Bioinforma.

[24] Allemeersch J, Durinck S, Vanderhaeghen R, Alard P, Maes R, Seeuws K, Bogaert T, Coddens K, Deschouwer K, Van Hummelen P (2005). Benchmarking the CATMA microarray. A novel tool for Arabidopsis transcriptome analysis. Plant Physiol.

[25] Girousse C, Bournoville R, Bonnemain J-L (1996). Water deficit-induced changes in concentrations in proline and some other amino acids in the phloem sap of alfalfa. Plant Physiol.

[26] Mäkelä P, Peltonen-Sainio P, Jokinen K, Pehu E, Setälä H, Hinkkanen R, Somersalo S (1996). Uptake and translocation of foliar-applied glycinebetaine in crop plants. Plant Sci.

[27] Lehmann S, Gumy C, Blatter E, Boeffel S, Fricke W, Rentsch D (2011). *In planta* function of compatible solute transporters of the AtProT family. J Exp Bot.

[28] Foster J, Lee YH, Tegeder M (2008). Distinct expression of members of the LHT amino acid transporter family in flowers indicates specific roles in plant reproduction. Sex Plant Reprod.

[29] Ábrahám E, Rigó G, Székely G, Nagy R, Koncz C, Szabados L (2003). Light-dependent induction of proline biosynthesis by abscisic acid and salt stress is inhibited by brassinosteroid in Arabidopsis. Plant Mol Biol.

[30] Guan Y, Meng X, Khanna R, LaMontagne E, Liu Y, Zhang S (2014). Phosphorylation of a WRKY transcription factor by MAPKs is required for pollen development and function in Arabidopsis. PLoS Genet.

[31] Lei R, Li X, Ma Z, Lv Y, Hu Y, Yu D (2017). Arabidopsis WRKY2 and WRKY34 transcription factors interact with VQ20 protein to modulate pollen development and function. Plant J.

[32] Wilkinson JE, Twell D, Lindsey K (1997). Activities of CaMV 35S and nos promoters in pollen: implications for field release of transgenic plants. J Exp Bot.

[33] Ariizumi T, Amagai M, Shibata D, Hatakeyama K, Watanabe M, Toriyama K (2002). Comparative study of promoter activity of three anther-specific genes encoding lipid transfer protein, xyloglucan endotransglucosylase/hydrolase and polygalacturonase in transgenic *Arabidopsis thaliana*. Plant Cell Rep.

[34] Costa-Nunes JA (2013). A novel Arabidopsis marker line that strongly labels uninucleate microspores and the subsequent male gametophyte development stages. Springerplus.

[35] Honys D, Twell D (2004). Transcriptome analysis of haploid male gametophyte development in Arabidopsis. Genome Biol.

[36] Hesse M, Pacini E, Willemse M (2012). The tapetum: cytology, function, biochemistry and evolution.

[37] Zhang HQ, Croes AF (1983). Proline metabolism in pollen - degradation of proline during germination and early tube growth. Planta.

[38] Yoshiba Y, Nanjo T, Miura S, Yamaguchi-Shinozaki K, Shinozaki K (1999). Stress-responsive and developmental regulation of *Delta(1)-pyrroline-5-carboxylate synthetase 1* (*P5CS1*) gene expression in *Arabidopsis thaliana*. Biochem Biophys Res Commun.

[39] Zou J, Song L, Zhang W, Wang Y, Ruan S, Wu WH (2009). Comparative proteomic analysis of Arabidopsis mature pollen and germinated pollen. J Integr Plant Biol.

[40] Holmes-Davis R, Tanaka CK, Vensel WH, Hurkman WJ, McCormick S (2005). Proteome mapping of mature pollen of Arabidopsis thaliana. Proteomics.

[41] Noir S, Brautigam A, Colby T, Schmidt J, Panstruga R (2005). A reference map of the Arabidopsis thaliana mature pollen proteome. Biochem Biophys Res Commun.

[42] Ischebeck T, Valledor L, Lyon D, Gingl S, Nagler M, Meijon M, Egelhofer V, Weckwerth W (2014). Comprehensive cell-specific protein analysis in early and late pollen development from diploid microsporocytes to pollen tube growth. Mol Cell Proteomics.

[43] Rotsch AH, Kopka J, Feussner I, Ischebeck T (2017). Central metabolite and sterol profiling divides tobacco male gametophyte development and pollen tube growth into eight metabolic phases. Plant J.

[44] Lehmann S, Funck D, Szabados L, Rentsch D (2010). Proline metabolism and transport in plant development. Amino Acids.

[45] Bernsdorff F, Döring A-C, Gruner K, Schuck S, Bräutigam A, Zeier J (2016). Pipecolic acid orchestrates plant systemic acquired resistance and defense priming via salicylic acid-dependent and -independent pathways. Plant Cell.

[46] Hellmann H, Funck D, Rentsch D, Frommer WB (2000). Hypersensitivity of an Arabidopsis sugar signaling mutant toward exogenous proline application. Plant Physiol.

[47] Návarová H, Bernsdorff F, Döring AC, Zeier J (2012). Pipecolic acid, an endogenous mediator of defense amplification and priming, is a critical regulator of inducible plant immunity. Plant Cell.

[48] Wang G, Zhang J, Wang G, Fan X, Sun X, Qin H, Xu N, Zhong M, Qiao Z, Tang Y (2014). Proline responding1 plays a critical role in regulating general protein synthesis and the cell cycle in maize. Plant Cell.

[49] Karimi M, Inze D, Depicker A (2002). GATEWAY vectors for *Agrobacterium*-mediated plant transformation. Trends Plant Sci.

[50] Earley KW, Haag JR, Pontes O, Opper K, Juehne T, Song K, Pikaard CS (2006). Gateway-compatible vectors for plant functional genomics and proteomics. Plant J.

[51] Clough SJ, Bent AF (1998). Floral dip: a simplified method for *Agrobacterium*-mediated transformation of *Arabidopsis thaliana*. Plant J.

[52] Bates LS, Waldren RP, Teare ID (1973). Rapid determination of free proline for water-stress studies. Plant Soil.

[53] Schneider CA, Rasband WS, Eliceiri KW (2012). NIH image to ImageJ: 25 years of image analysis. Nat Methods.

[54] Cecchetti V, Brunetti P, Napoli N, Fattorini L, Altamura MM, Costantino P, Cardarelli M (2015). ABCB1 and ABCB19 auxin transporters have synergistic effects on early and late Arabidopsis anther development. J Integr Plant Biol.

[55] Francioso A, Fanelli S, Vigli D, Ricceri L, Cavallaro RA, Baseggio Conrado A, Fontana M, D'Erme M, Mosca L (2017). HPLC determination of bioactive sulfur compounds, amino acids and biogenic amines in biological specimens. Adv Exp Med Biol.

[56] Jefferson RA, Kavanagh TA, Bevan MW (1987). GUS fusions: beta-glucuronidase as a sensitive and versatile gene fusion marker in higher plants. EMBO J.

[57] Plegt L, Bino RJ (1989). ß-glucuronidase activity during development of the male gametophyte from transgenic and non-transgenic plants. Mol Gen Genet.

[58] Hu CY, Chee PP, Chesney RH, Zhou JH, Miller PD, O'Brien WT (1990). Intrinsic GUS-like activities in seed plants. Plant Cell Rep.

[59] Nishihara M, Ito M, Tanaka I, Kyo M, Ono K, Irifune K, Morikawa H (1993). Expression of the ß-glucuronidase gene in pollen of lily (*Lilium longiflorum*), tobacco (*Nicotiana tabacum*), Nicotiana rustica, and Peony (*Paeonia lactiflora*) by particle bombardment. Plant Physiol.

