# Supplementary material for: Proline synthesis in developing microspores is required for pollen development and fertility
Source: BMC Plant Biol. 2018 Dec 17;18:356. doi: 10.1186/s12870-018-1571-3 (PMC6296085; doi:10.1186/s12870-018-1571-3)
Supplement: Supplementary file 1 — Figure S1. Histochemical localization of GUS activity in anthers of pP5CS1:GUS and pP5CS2:GUS transgenic Arabidopsis plants. Inflorescences of pP5CS1:GUS (A-C) and pP5CS2:GUS (D-F) transgenic plants were infiltrated with X-Gluc solution, stained overnight, fixed and cleared for microscopic analysis. Each panel shows a whole-mount anther at stage 12/13 from an independent transgenic line. GUS activity was exclusively detected in pollen grains. Scale bars are 50 μm in A, B, D, E and 25 μm in C and F. (PDF 2779 kb) [file 12870_2018_1571_MOESM1_ESM.pdf]

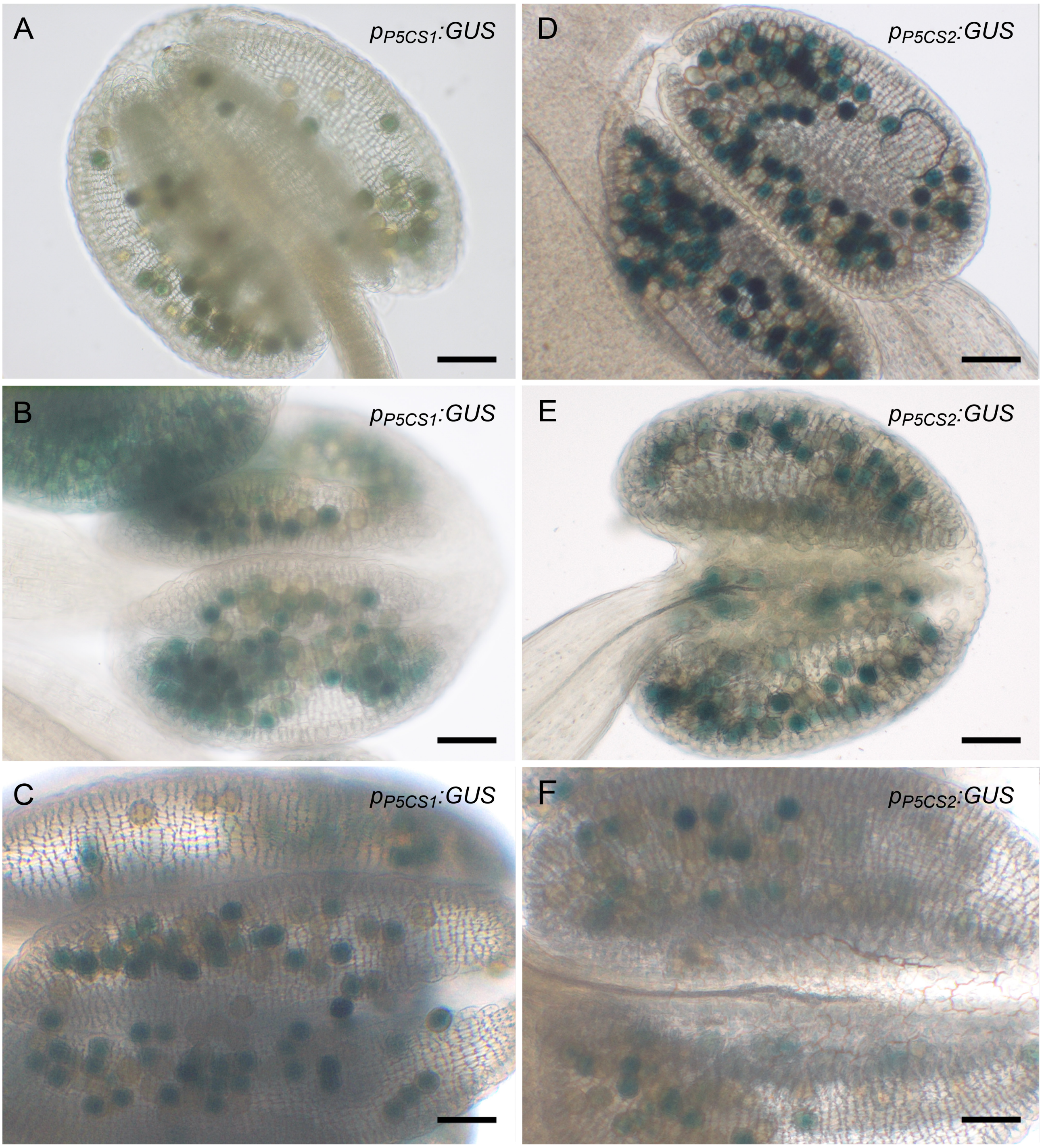

Additional file 1: Figure S1. Histochemical localization of GUS activity in anthers of *p_{P5CS1}:GUS*, and *p_{P5CS2}:GUS* transgenic Arabidopsis plants.

Inflorescences of *p_{P5CS1}:GUS* **(A-C)** and *p_{P5CS2}:GUS* **(D-F)** transgenic plants were infiltrated with X-Gluc solution, stained overnight, fixed and cleared for microscopic analysis. Each panel shows a whole-mount anther at stage 12/13 from an independent transgenic line. GUS activity was exclusively detected in pollen grains. Scale bars are 50 µm in A, B, D, E and 25 µm in C and F.
