# Supplementary material for: Proline synthesis in developing microspores is required for pollen development and fertility
Source: BMC Plant Biol. 2018 Dec 17;18:356. doi: 10.1186/s12870-018-1571-3 (PMC6296085; doi:10.1186/s12870-018-1571-3)
Supplement: Supplementary file 2 — Figure S2. Predicted cis-regulatory elements in the promoters of P5CS1 and P5CS2. Schematic map outlining the main putative binding sites for transcription factors derived from a PlantPAN2 (http://plantpan2.itps.ncku.edu.tw) and PLACE (http://www.dna.affrc.go.jp/PLACE/) in silico analysis of P5CS1 (At2g39800) and P5CS2 (At3g55610) promoters. The promoter analysis was carried out on 2932 bp and 2097 bp upstream of the start codons of either P5CS1 or P5CS2, respectively. Putative cis-regulatory elements corresponding to binding motifs of transcription factors related to pollen development and fertility (SBP, bHLH, WRKY; GO terms “associated with pollen development” [GO:0009555], “pollen tube growth” [GO:0009860], “anther development” [GO:00048643] and “double fertilization forming a zygote and endosperm” [GO:0009567]) are significantly enriched and highlighted in red. (PDF 1149 kb) [file 12870_2018_1571_MOESM2_ESM.pdf]

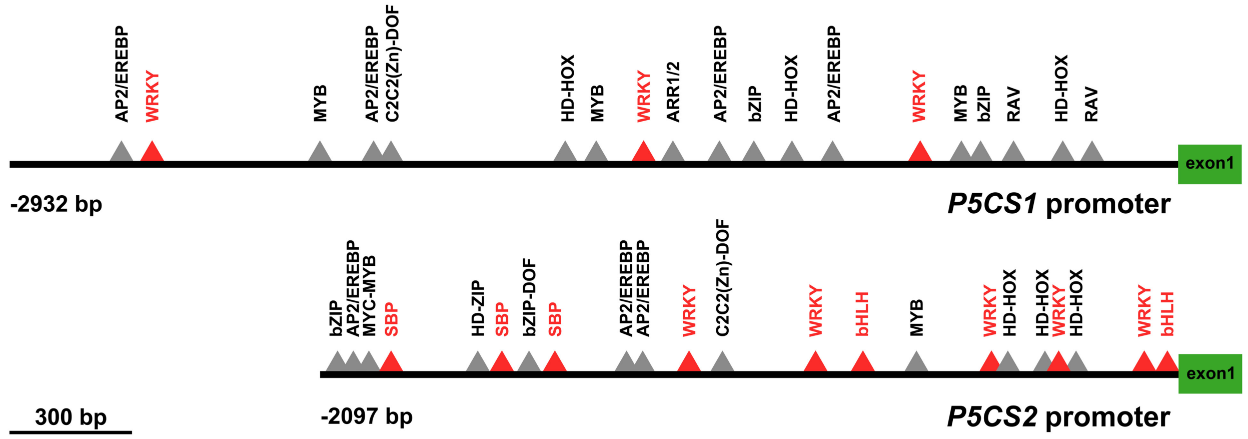

Additional file 2:

Figure S2. Predicted *cis*-regulatory elements in the promoters of *P5CS1* and *P5CS2.*

Schematic map outlining the main putative binding sites for transcription factors derived from a PlantPAN2 (http://plantpan2.itps.ncku.edu.tw) and PLACE (http://www.dna.affrc.go.jp/PLACE/) *in silico* analysis of *P5CS1* (*At2g39800*) and *P5CS2* (*At3g55610*) promoters. The promoter analysis was carried out on 2932 bp and 2097 bp upstream of the start codons of either *P5CS1* or *P5CS2*, respectively. Putative cis-regulatory elements corresponding to binding motifs of transcription factors related to pollen development and fertility (SBP, bHLH, WRKY; GO terms “associated with pollen development” [GO:0009555], “pollen tube growth” [GO:0009860], “anther development” [GO:00048643] and “double fertilization forming a zygote and endosperm” [GO:0009567]) are significantly enriched and highlighted in red.
