# Supplementary material for: Proline synthesis in developing microspores is required for pollen development and fertility
Source: BMC Plant Biol. 2018 Dec 17;18:356. doi: 10.1186/s12870-018-1571-3 (PMC6296085; doi:10.1186/s12870-018-1571-3)
Supplement: Supplementary file 3 — Figure S3. Histochemical localization of GUS activity in anthers of p35S:GUS, pLtp12:GUS and p17340:GUS transgenic Arabidopsis plants. Inflorescences of p35S:GUS (A,B), pLtp12:GUS (C,D) and p17340:GUS (E,F) transgenic plants were infiltrated with X-Gluc solution, stained overnight, fixed and cleared for microscopic analysis. Each panel shows a whole-mount anther at stage 12/13 (A,B,E,F) or stage 9/10 (C,D) from an independent transgenic line. GUS activity was detected in the filaments and vascular tissues of p35S:GUS anthers but not in pollen grains. The pLtp12:GUS construct induced GUS activity specifically in the tapetum, whereas GUS activity in p17340:GUS transgenic anthers was almost exclusively detected in pollen grains. Scale bars are 50 μm. (PDF 2798 kb) [file 12870_2018_1571_MOESM3_ESM.pdf]

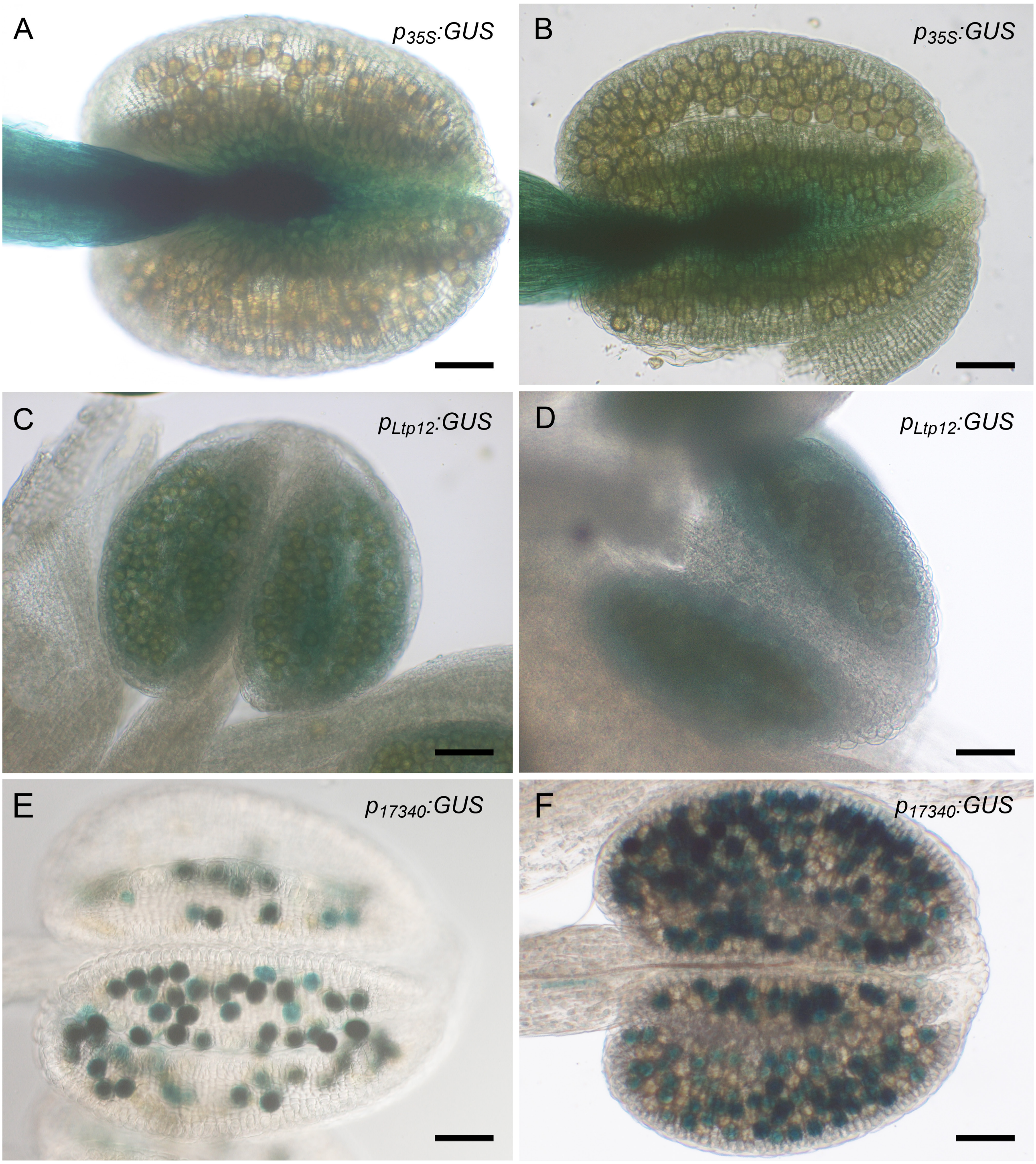

Additional file 3: Figure S3. Histochemical localization of GUS activity in anthers of *p_{35S}:GUS*, *p_{Ltp12}:GUS* and *p_{17340}:GUS* transgenic Arabidopsis plants.

Inflorescences of *p_{35S}:GUS* (**A**,**B**), *p_{Ltp12}:GUS* (**C**,**D**) and *p_{17340}:GUS* (**E**,**F**) transgenic plants were infiltrated with X-Gluc solution, stained overnight, fixed and cleared for microscopic analysis. Each panel shows a whole-mount anther at stage 12/13 (**A**,**B**,**E**,**F**) or stage 9/10 (**C**,**D**) from an independent transgenic line. GUS activity was detected in the filaments and vascular tissues of *p_{35S}:GUS* anthers but not in pollen grains. The *p_{Ltp12}:GUS* construct induced GUS activity specifically in the tapetum, wheras GUS activity in *p_{17340}:GUS* transgenic anthers was almost exclusively detected in pollen grains. Scale bars are 50 µm.
