# Supplementary material for: Proline synthesis in developing microspores is required for pollen development and fertility
Source: BMC Plant Biol. 2018 Dec 17;18:356. doi: 10.1186/s12870-018-1571-3 (PMC6296085; doi:10.1186/s12870-018-1571-3)
Supplement: Supplementary file 4 — Table S1. Overview of pollen morphology and fertility data. (PDF 22 kb) [file 12870_2018_1571_MOESM4_ESM.pdf]

Additional file 4: **Table S1:** Overview of pollen morphology and fertility data

Arabidopsis line	Col-0	*p5cs*	*p5cs* + p_{35S}:*P5CS2*	*p5cs* + p_{Ltp12}:*P5CS2*^{m}	*p5cs* + p_{Ltp12}:*P5CS2*^{s}	*p5cs* + p_{17340}:*P5CS2* het	*p5cs* + p_{17340}:*P5CS2* hom
aberrant pollen grains [%][a] from Fig.3	nd (7;74)	51.5±3.6 (6;52)	46.6±7.4 (6;81)				
aberrant pollen grains + NaCl [%][a] from Fig.3	nd (10;88)	48.9±5.6 (9;187)	46.1±5.3 (42;311)				
aberrant pollen grains [%][a] from Fig.5	nd (11;104)	46.2±3.3 (5;384)		30.9±1.2 (10;935)	17.8±2.4 (12;2505)		
aberrant pollen grains [%][a] from Fig.6	nd (8;96)	42.6±1.3 (16;535)				19.5±1.4 (40;1959)	0.2±0.1 (22;1527)
aberrant seeds after selfing [%][a]	nd (3;113)	nd (5;197)	nd (5;222)				
aberrant seeds after selfing + NaCl [%][a]	nd (4;174)	nd (5;190)	nd (6;250)				
aberrant seeds after selfing [%][a] from Fig.5	nd (6;246)	nd (10;440)		5.4±1.1 (7;291)	6.2±0.9 (33;1293)		
aberrant seeds after selfing [%][a] from Fig.6	nd (3;124)	nd (3;172)				12.1±0.8 (9;431)	24.6±2.4 (9;327)
p5cs2-1 transmisson to Col-0 [%][a,d]	-:-	nd (5;172)	nd (2;65)				
p5cs2-1 transmisson to Col-0 [%][a] from Fig.5	-:-	nd (4;120)		15.0±5.0 (2;20)[e]	14.6±2.3 (21;1289)		
p5cs2-1 transmisson to Col-0 [%][a] from Fig.7	-:-	nd (4;115)				26.3±1.9 (3;182)	45.7±0.5 (3;94)
proline in inflorescences [ng/mg fw][a] from Fig.3	162.2±15.9 (5;100)	25.6±2.2 (5;100)	23.4±1.1 (5;100)				
proline in inflorescences + NaCl [ng/mg fw][a] from Fig.3	964.6±75.8 (5;100)	21.6±1.1 (5;100)	145.2±7 (5;100)				
proline in pollen [pg/grain][a] from Fig.8	38.9±2.4 (3;~3000)	14.4±0.6 (3;~3000)	15.0±0.6 (3;~3000)	19.9±1.6 (3;~3000)	na	na	47.0±4.6 (3;~3000)
proline in anthers [ng/anther][a] from Fig.8	35.6±2.2 (2;~400)	12.6±1.0 (2;~400)	32.3±1.2 (2;~400)	18.2±3.1 (2;~400)	na	na	13.0±2.5 (2;~400)
fertile *p5cs1/p5cs2* pollen [%] from selfing[b]	-:-	3.4	0	21.6	24.8	48.4	98.4
fertile *p5cs1/p5cs2* pollen [%] from crossing[c]	-:-	0	0	30.0	29.2	52.6	91.4

a all data are mean±SE (number of independent samples;total N)
b calculated as X/25*100 = Nr of observed aborted seeds/Nr of expected aborted seeds*100
c calculated as X/50*100 = Nr of observed *p5cs2-1* heterozygous seedlings/Nr of expected *p5cs2-1* heterozygous seedlings*100
d treated with 0.1 M NaCl
e analyzed by PCR
nd not detected
na not analyzed
