# Supplementary material for: Proline synthesis in developing microspores is required for pollen development and fertility
Source: BMC Plant Biol. 2018 Dec 17;18:356. doi: 10.1186/s12870-018-1571-3 (PMC6296085; doi:10.1186/s12870-018-1571-3)
Supplement: Supplementary file 5 — Table S2. Primers used in this study (PDF 35 kb) [file 12870_2018_1571_MOESM5_ESM.pdf]

Additional file 5: Table S2: Primers used in this study

Sequence	Name	purpose
ataaagcttttcgaggcgtatgcgttcac	proP5CS1_HindIII-f	*P5CS1* promoter for insertion in pBI121
ataggatcctcgtcgtcgtcgtctaccaaa	proP5CS1_BamHI-r	
ataaagctttgggatacatgatttcactatgtgtg	proP5CS2_HindIII-f	*P5CS2* promoter for insertion in pBI121
ataggatcctcgtcgtcgtcgtctaccaaa	proP5Cs2_BamHI-r	
gaccatgattacgccaagcttTATGAAGAAAGATTCGGATTCAAG	pro17340-f	*17340* promoter for insertion in pBI121
ccacccggggatcctctagaTTTTGGATTTTTTAGGAAACTTTTG	pro17340-r	
gtCCCgggTTCGGAGTAAACGCCATttttg	proLtp12_Sma-r	*Ltp12* promoter for insertion in pHGWFS7
caccgagctcactagTtcgatttacgattatac	proLtp12_SacSpe-f	
ggcgtttcctggtttgataac	proP5CS1_GUS-f	Genotyping of p_{P5CS1}:*GUS* transgenic plants
GGCACAGCACATCAAAGAGAT	proP5CS1_GUS-r	
tgctaagaacaaccgacaacaaaa	proP5CS2_GUS-f	Genotyping of p_{P5CS2}:*GUS* transgenic plants
CCAGCCATGCACACTGATAC	proP5CS2_GUS-r	
AAACCTCCTCGGATTCCATTG	35S-f	Genotyping of pBI121 transgenic palnts
GCAATTGCCCGGCTTTCTTGTAAC	GUS-5'-r	Genotyping of *GUS* transgenic plants
ataggatccATGACGGAGATCGATCGTTC	P5CS2_BamHI-f	*P5CS2* cloning in pBI121
gaagagctcCTAAATTCCATTCTCAACAGC	P5CS2_SacI-r1	
caccATGgCGGAGATCGATCGTTCAC	P5CS2_Nco-Ef	*P5CS2* cloning in pENTR
aagagctcaCTAAATTCCATTCTCAACAGC	P5CS2_SacI-r2	
ATCGATCTCCGcCATgATCTTCGGAGTAAACGCCA	proLtp12_P5CS2-r	*Ltp12* promoter for pENTR-*P5CS2*
gcccccttcaccatgtgtagtgtgttttccaagca	proLtp12_pENTR-f	
AAACCTCCTCGGATTCCATTG	17340_P5CS2-f	p_{17340}:*P5CS2* and p_{17340}:*GUS* detection
TTGCCATGTCCAGTTTACCAGA	17340_P5CS2-r	p_{17340}:*P5CS2* detection
CTGTTGGGGGTAAACTCATTG	P5CS1-f	*P5CS1*: gene detection
CTGTTGGGGGTAAACTCATTG	P5CS1-r	*P5CS1*: gene detection
GCGTGGACCGCTTGCTGCAACT	LBb1-r	*P5CS1*:T-DNA junction detection
GGAGCAGAATGGTTTTCTCG	P5CS2-f	*P5CS2*: gene detection
TGGAAAACAGCAGCActgtc	P5CS2-r	*P5CS2*: gene detection
tatctgggaatggcgaaatc	Gabi-LB-r	*P5CS2*:T-DNA junction detection
CAAGCAATGGTGGAAGAGTAAA	Sul-f	detection of *sul* resistance gene
CGGGGCTCAAGAAAAATCC	Sul-r	
